# Advancements in Targeting Macrophage Senescence for Age-Associated Conditions

**DOI:** 10.14336/AD.2024.0720

**Published:** 2025-09-14

**Authors:** Jingwei Xiao, Hung Sing Li, Senthil Kumaran Satyanarayanan, Shu Lai Leung, Qiuju Yuan, Yaofeng Wang, Dajiang Qin, Suki Man Yan Lee

**Affiliations:** ^1^Centre for Regenerative Medicine and Health, Hong Kong Institute of Science & Innovation, Chinese Academy of Sciences, Hong Kong SAR, China.; ^2^Key Laboratory of Biological Targeting Diagnosis, Therapy and Rehabilitation of Guangdong Higher Education Institutes, The Fifth Affiliated Hospital of Guangzhou Medical University, Guangzhou, China.; ^3^School of Chinese Medicine, Faculty of Medicine, The Chinese University of Hong Kong, Hong Kong SAR, China.; ^4^Bioland Laboratory, Guangzhou Regenerative Medicine and Health Guangdong Laboratory, Guangzhou, China.; ^5^School of Public Health, Li Ka Shing Faculty of Medicine, The University of Hong Kong, Hong Kong SAR, China.

**Keywords:** Inflammaging, Macrophage Senescence, Chronic Inflammation, Senescence-Associated Secretory Phenotype (SASP), Innate Immune Signaling

## Abstract

Macrophages, a critical subset of innate immune cells, play a pivotal role in cytokine production during disease progression, tissue injury, and pathogen invasion. Their intricate involvement in the manifestation of chronic low-grade inflammation associated with the aging process is widely acknowledged. Notably, in aged tissues, macrophages exhibit an altered phenotype characterized by an augmented synthesis of pro-inflammatory cytokines and chemokines, a profile intimately associated with a phenomenon known as inflammaging. Macrophages possess the capacity to undergo cellular senescence, a state of permanent growth arrest, in response to diverse stressors, including aging. Senescent macrophages secrete an array of pro-inflammatory molecules, growth factors, and matrix metalloproteinases, collectively referred to as the Senescence-Associated Secretory Phenotype (SASP). The SASP exacerbates the state of chronic inflammation observed in aging tissues. Thus, disruptions in macrophage function and signaling pathways due to aging result in escalated production of inflammatory mediators, perpetuating inflammaging. Recent research has uncovered novel mechanisms centred around innate immune signaling and mitochondrial dysfunction in macrophages, highlighting their crucial role in the development of inflammaging and associated pathological conditions. This review delves into the latest scientific findings on these emerging mechanisms in macrophage senescence related to aging and explores the prospects of targeting macrophages to address age-associated conditions effectively.

## 1. Introduction

Macrophages are effector cells of the innate immune system present in all tissues under physiological homeostatic conditions [1]. They play a critical role in removing pathogens and dead cells through phagocytosis, inducing inflammatory responses, mediating immune responses, promoting tissue repair and regeneration, and regulating host homeostasis [2]. In addition, macrophages demonstrate remarkable plasticity, enabling them to reversibly transition between different phenotypes, such as pro-inflammatory and anti-inflammatory macrophages [3, 4]. This ability positions them as key players in the spectrum of chronic inflammation [1]. Inappropriate activation or inflammation responses can interrupt the homeostatic function of macrophages, resulting in uncontrollable inflammatory responses and leading to tissue or organ damage, which is related to the progression of chronic diseases [5]. Recent studies have highlighted the key role of macrophages in the process of inflammaging [6], a concept introduced by Franceschi et al. that describes profound alterations in the immune systems during aging [7]. This age-related immune dysregulation has been demonstrated as a primary contributor to inflammaging, leading to homeostatic frailty and immune debilitation in the elderly [8].

Aging is a complex and irreversible pathophysiological process that results in declines in tissue and cell functions, causing various age-related pathologies such as cardiovascular diseases, neurodegenerative diseases, and immune system diseases [9, 10]. Several complex and important pathways drive the aging process, and many molecular mechanisms modulating aging have been reported, including loss of proteostasis, telomere dysfunction, mitochondrial dysfunction, epigenetic alterations, and stem cell exhaustion [10, 11]. During aging, macrophages undergo senescence, becoming dysfunctional and losing their ability to carry out their normal functions effectively [12, 13], where cellular senescence significant impacts tissue homeostasis maintenance under normal and pathological conditions and has been demonstrated as a principal causative factor in aging and facilitates the progression of aging and aging-related diseases [10, 14]. In addition, there is a shift in the balance of macrophage subtypes, characterized by pro-, anti-inflammatory states [15, 16]. Additionally, the phagocytic and chemotactic functions of macrophages are altered in aging. In aged mice, peritoneal and brain-resident macrophages exhibit reduced phagocytosis of myelin fragments, fluorescent particles, apoptotic neutrophils, and lipid debris [17]. Targeting macrophages in age-related diseases presents a promising therapeutic approach for restoring their phagocytic function and modulating their cytokine secretion profile, which, in turn, may offer potential benefits for tissue repair or rejuvenation [18]. Aging is the main risk factor for most neurodegenerative diseases, including Alzheimer's disease (AD), Huntington's disease, and Parkinson's disease (PD) [19, 20]. The accumulation of dysfunctional and senescent macrophages in the central nervous system (CNS) can impair phagocytosis, alter cytokine profile, impair neuroprotection, and compromise the blood-brain barrier (BBB), suggesting that regulating macrophages could improve the treatments for age-associated CNS diseases [21, 22]. Macrophages exhibit a high degree of heterogeneity and possess epigenomes that demonstrate substantial plasticity [23]. Their phenotypic plasticity allows for dynamic transcriptional reprogramming in response to changes in the surrounding tissue microenvironment, leading to functional modifications of macrophages [24].

To enhance the drug-targeting specificity of macrophages and their subtypes in tissues, macrophage-targeted micro-based therapeutics have been developed for treating various diseases [5, 10]. These therapeutics can deliver drugs or other therapeutic agents directly to macrophages by targeting macrophage surface receptors or incorporating with exosomes to enhance the recognition, enabling more efficient and targeted treatment [25]. However, several challenges remain before these therapeutics can be used in clinical settings, including issues of delivery efficiency, efficacy, and safety. In this review, we explore the physiological roles and functions of macrophages, as well as the concept of macrophage plasticity, polarization, and senescence in the context of age-related diseases, with particular emphasis on the association between mitochondrial dysfunction and aging.

We further summarized the current macrophage-targeted micro-based therapeutics in age-related diseases, particularly focusing on CNS-associated diseases. This includes highlighting examples of macrophage-targeted delivery systems and identifying current challenges that need to be addressed prior clinical use. Targeting macrophages as a therapeutic approach offers promising prospects for developing effective treatments for age-related disease treatments.

## 2. Role and function of macrophage in healthy and disease state CNS

Macrophages serve as a vital component in the immune system [26]. As professional phagocytes, they play prominent roles in immune surveillance, the maintenance of immune homeostasis, and the orchestration of inflammatory responses [27]. Furthermore, macrophages contribute to the establishment of immunological memory, the facilitation of tissue repair, and various developmental processes. This broad range of functions is enabled by its remarkable plasticity that allows the dynamic adjustment of their phenotypes in response to the diverse microenvironmental cues. Through their strategic positioning at the interface of innate and adaptive immunity, as well as their functional versatility, macrophages emerge as central regulators of immune homeostasis, inflammation, and tissue homeostasis [28, 29]. Macrophages may exhibit a remarkable degree of tissue-specific specialization, with distinct subtypes residing in various organs and exhibiting unique functional characteristics. For instance, Kupffer cells in the liver, alveolar macrophages in the lung, osteoclasts in bone, and Langerhans cells in the skin each play specialized roles in maintaining organ-specific homeostasis and coordinating diverse physiological processes [30, 31]. These macrophages are relatively motile, phenotypically plastic, and longevous in nature; they are thus considered as the potential targets in modulating disease processes. Similar to the tissue-resident macrophages found in other organs, the macrophages within CNS also exhibit heterogeneity, with distinct patterns of gene expression and morphological characteristics.

Anatomically, macrophages residing in the CNS are typically categorized into two types: microglia located at the CNS parenchyma and non-parenchymal border-associated macrophages (BAM) [32]. Recent research employing high-dimensional analytical techniques has revealed the detailed subpopulations of these macrophages and elucidated their functions in CNS homeostasis and disease processes. In the subsequent sections, we will provide an overview of the known roles and functions of these macrophage subtypes and examine the influence of cellular senescence on their activities.

### 2.1 The development and functions of macrophages in CNS under homeostatic conditions

#### 2.1.1 Microglia

In the adult brain, microglia are the key immune cells found at the parenchyma, which accounts for about ten percent of the total cells and is locally the most abundant phagocyte [29]. Microglial cells can be detected at the 4.5 gestational weeks and enter the CNS in humans [33]. It has been unveiled that the macrophages in the CNS, such as microglia and BAMs, are derived from the same origin [34-36]. A previous study using a mouse model demonstrated that they emerged from the c-Kit^+^ TLR2^+^ precursors of erythromyeloid progenitors (EMP) in the embryonic yolk sac at embryonic day 7.5 (E7.5) [37]. Another study reported that microglia originated from the yolk sac primitive c-kit^+^ EMP and differentiated into CD45^+^ CX3CR1^+^ c-kit^-^ cells (A2) through the intermediate CD45^+^ CX3CR1^-^ c-kit^lo^ immature population (A1) [38]. Two subpopulations were later identified from the A2 cells using scRNA-seq and flow cytometry; the A2 subset expressing CD206 (CD206^+^ A2) belongs to the BAM progenitors, while the CD206^-^ A2 cells are the microglia progenitor [39]. These macrophage progenitors then migrate to the neural tube *via* the developing blood vessels, and the CD206^-^ A2 cells seeding the parenchyma later differentiate into microglia [35, 40]. The microglial cell population in the brain is maintained by self-renewal in which the microglia expansion process under steady-state conditions is carried out randomly [37, 41].

Utilizing bulk and single-cell RNA sequencing (scRNA-seq) technologies, the transcriptional profile of microglia were found evolutionarily conserved [42]. This conservation implies that microglial functions are comparable across different species. Microglia play indispensable roles in various cellular processes and facilitate communication between glial cells and neurons. During embryogenesis, microglia contribute to vasculogenesis, including the formation of anastomoses and vascular sprouting [43]. Microglia participate in the establishment of neuronal circuits and modulate neurogenesis via regulating the amount of neuronal progenitor cells (NPCs); they also help to engulf and clear apoptotic neurons [31, 44]. Microglia are important in modulating synaptic plasticity and transmission [39, 45]. During postnatal development, microglia shape adolescent neuronal circuits through apoptosis-coupled phagocytosis in the neurogenic regions of the hippocampus [46]. In addition, they produce insulin-like growth factor-1 (IGF-1), a trophic factor that signals the survival of layer V cortical neurons and promotes the development of oligodendrocyte progenitor cells (OPC) [47, 48]. In adult brain, microglia participate in myelin sheath homeostasis partly through Rab7-mediated phagocytosis [49]. Under homeostatic conditions, microglia remain highly active, they survey the microenvironment continuously with protrusions and motile processes [50]. More importantly, microglia are responsible for monitoring neuronal functions through direct physical interactions between their processes and cell bodies of the neurone [51].

#### 2.1.2 Border-associated macrophages

BAMs are located at the CNS interfaces, encompassing the CNS border niches, including meninges, perivascular space, and choroid plexus. The BAM population is highly heterogeneous, with distinct subsets defined by their specific anatomical localization within the CNS borders. These subsets include dural macrophages (dmMΦ), leptomeningeal macrophages (MMΦ), perivascular macrophages (PVMΦ), and choroid plexus macrophages (cpMΦ) [31, 52]. Traditionally, it was believed that BAMs originated from bone-marrow-derived monocytes postnatally, characterized by their short lifespan and high turnover [53]. However, recent data indicate that BAMs and microglia likely share a common origin [34, 35]. Research by Sankowski et al. revealed that these cells diverge in transcriptional profiles as early as five weeks post-conception [54]. It was also previously thought that all BAMs originate prenatally from the yolk sac and fetal liver-derived progenitors during embryonic development, seeding the CNS interfaces and differentiating within their respective niches [35, 39, 55]. However, latest findings demonstrate that meningeal macrophages are the only population that derived from the same prenatal progenitor as microglia, which later postnatally populate the perivascular space and establish into PVMΦ in the presence of integrin [56].

Phenotypically, the MHCII^-^ CCR2^-^ CD38^+^ sub-population contribute to approximately 75% of the BAM, and the MHCII^+^ CD38^+^ sub-population are predominantly composed of PVMs and MMΦ [57]. Regarding the maintenance of BAMs, PVMΦ and MMΦ do not primarily rely on monocyte recruitment from the peripheral circulation. These BAM subsets are relatively long-lived and capable of self-renewal with minimal turnover, suggesting that their population homeostasis is largely sustained via *in situ* proliferation instead of continuous replenishment from the bone marrow [29, 39, 58]. On the other hand, the dmMΦ have a more complex ontogeny. They can be subdivided into two distinct subsets: an MHCII^-^ subset that emerges during embryonic development and is long-lived and an MHCII^+^ subset that is potentially derived from bone marrow-derived monocytes [39, 55]. This aligns with a study that identified the bone marrow niche adjacent to the spinal cord and brain in maintaining the myeloid pool in the meninges under steady and disease states [59]. cpMΦ originates from primitive macrophages during embryogenesis and resides around the blood-CSF barrier. This population is maintained through the continuous replenishment of CCR2^+^ Ly6C^high^ monocytes from the peripheral circulation, resulting in a high turnover rate for cpMΦs compared to self-renewing BAM subpopulations [29, 55, 60].

Among the tissue-resident macrophages in the brain, the physiological function of BAMs is less extensively studied compared with microglia. BAMs regulate molecule exchange between peripheral tissues and the brain [58]. It is suggested that PVMΦ contribute to the maintenance of brain homeostasis both functionally and structurally under physiological conditions. They participate in immunity through phagocytosis and antigen presentation, help to maintain BBB integrity, and facilitate lymphatic drainage [61]. Transcriptional profiling analyses suggest that cpMΦ are involved in a range of functional processes, such as immune responses, phagocytosis, antigen presentation, and lipid metabolism [62]. Despite these insights, the unique population and corresponding functions of different BAM subtypes remain poorly understood. In addition, the rarity of BAMs in the CNS and the difficulty distinguishing them from other myeloid populations like microglia have hindered the comprehensive characterization of this heterogeneous compartment. Further in-depth research is required to shed light on the specialized roles of BAM subsets fully.

### 2.2 Heterogeneity of CNS macrophage in disease states

Microglia exhibit dysregulated states in neurodegenerative diseases [30]. Keren-Shaul et al. described a distinct microglial subpopulation, termed disease-associated microglia (DAM), displays altered gene expression and functional characteristics in the brains of mouse models and patients with AD [39, 63, 64]. As indicated in preclinical studies, this subset may play a protective role in the brain of human AD patients [63]. Interestingly, DAM-like cells have also been observed in aged, non-diseased mouse brains, although their functional significance remains unclear [57]. Using scRNA-seq, it has been revealed that DAMs exhibit higher expression of Cd9, Cd11c, and Csf1 compared with microglia, while the level of several microglial homeostatic genes, including Cx3cr1, Tmem119 and P2ry12/P2ry13, are reduced in DAMs [63]. Functionally, DAMs are capable of phagocytosing amyloid-beta (Aβ) aggregates and activating pathways that mediate immune responses [55, 58, 63, 64].

The transition to a DAM phenotype involves a two-stage activation process, with the triggering receptor expressed on myeloid cell 2 (TREM2) playing a crucial role in the second phase. Initially, DAMs enter a primed state characterized by promoting gene expression involved in immune response, phagocytosis, and lipid metabolism. However, the full induction of these functional programs requires the TREM2-dependent second activation phase [63]. The loss of TREM2 is linked to various neurodegenerative diseases. For instance, loss-of-function mutation in TREM2 leads to dementia [65], and a rare R47H mutation in TREM2 is a risk factor for developing AD [66]. These findings are consistent with the observations that TREM2 is absent in microglia during the late stages of AD which exacerbates disease progression [63, 66]. Thus, the full activation of DAMs is dependent on the expression and signaling of functional TREM2 [63].

Using a global myeloid cell map known as M-Verse, Silvin et al. further refined two distinct macrophage lineages in AD mouse models that differ in their function and ontogeny [64]. The M-Verse incorporated six scRNA-seq datasets from different studies which covers a wide range of CNS conditions, the diverse datasets enable the resolution of macrophage heterogeneity in mice brain. In the analysis, the *bona fide* DAMs were found in the developmental microglia area, indicating an embryonic origin. Conversely, the mature microglia area harbours another subset, known as disease-inflammatory macrophages (DIMs), originated from the peripheral monocytes. Although both DAMs and DIMs express TREM2, the DIM subset is TREM2-independent. DAMs can also be distinguished from disease-inflammatory macrophages (DIMs) by the specific expression of genes such as Dkk2, Fabp5, Gm1673, Gpnmb, Igf1, Itgax, Mamdc2, and Spp1 [64]. Unlike the DAMs, DIMs appear and expand dramatically during aging and conditions like AD. Notably, DIMs were observed near Aβ plaques in AD mouse brains. Further analysis revealed that DIMs uniquely express genes primarily involved in pro-inflammatory pathways, such as interleukin-1 (IL-1), IL-6, TNF, Toll-like receptor signaling, nitric oxide production, and reactive oxygen species generation. This indicates that DIMs may play a distinct, potentially more inflammatory role in neurodegeneration associated with aging and neuropathology compared to the DAMs [64].

### 2.3 Macrophage and inflammaging

Aging encompasses a series of pathophysiological processes characterized by systemic chronic inflammation, immunosenescence and cellular senescence. This process is associated with deteriorating barriers such as skin, BBB, and gut mucosa [58]. Factors contributing to the progressive decline in tissue and cellular functions during aging include accumulated epigenetic changes and DNA damage, disruption of protein homeostasis, immune dysregulation, and lysosomal dysfunction [58]. The aging process is irreversible and often accompanied by an augmented risk of age-associated conditions at both cellular and organ levels, leading to chronic diseases in senile individuals such as AD, PD, atherosclerosis, non-alcoholic fatty liver disease, osteoporosis, age-related macular degeneration, and chronic obstructive pulmonary disease (COPD) [10, 67-69]. The detrimental consequence of aging on the immune system has been extensively studied. The term ‘Inflammaging’ describes the chronic, low-grade systemic inflammation associated with aging, likely linked to the accumulation of cell debris, immune-globulins, cell senescence, and immunosenescence due to persistent inflammatory stimulation [7, 9, 70]. Senescent cells produce a bioactive secretome known as the senescence-associated secretory phenotype (SASP), which mediates various pathophysiological processes and recruits immune cells. Macrophages, as critical components of the immune system, can be affected by SASP; their polarization and senescence can be induced by the surrounding senescent cells in a paracrine manner [71]. Disruptions in macrophage signaling and function can further elevate the production of pro-inflammatory cytokines, exacerbating age-related pathologies.

As the brain ages, myelin fragments are released from deteriorating myelin sheaths. Microglia clear these fragments, leading to the accumulation of lipofuscin-like insoluble lysosomal inclusions within these cells [49]. This accumulation results in a subtype of microglia known as lipid-droplet-accumulating microglia (LDAM), which exhibit altered energy regulation and cellular metabolism [72, 73]. LDAM is characterized by the elevated levels of reactive oxygen species (ROS), production of pro-inflammatory cytokines, and impaired phagocytic abilities [58, 72, 73]. These studies unveiled significant shifts in gene expression and cellular metabolism in aging macrophages, some of which are hallmarks of cellular senescence.

## 3. Macrophage senescence

**Figure 1.**
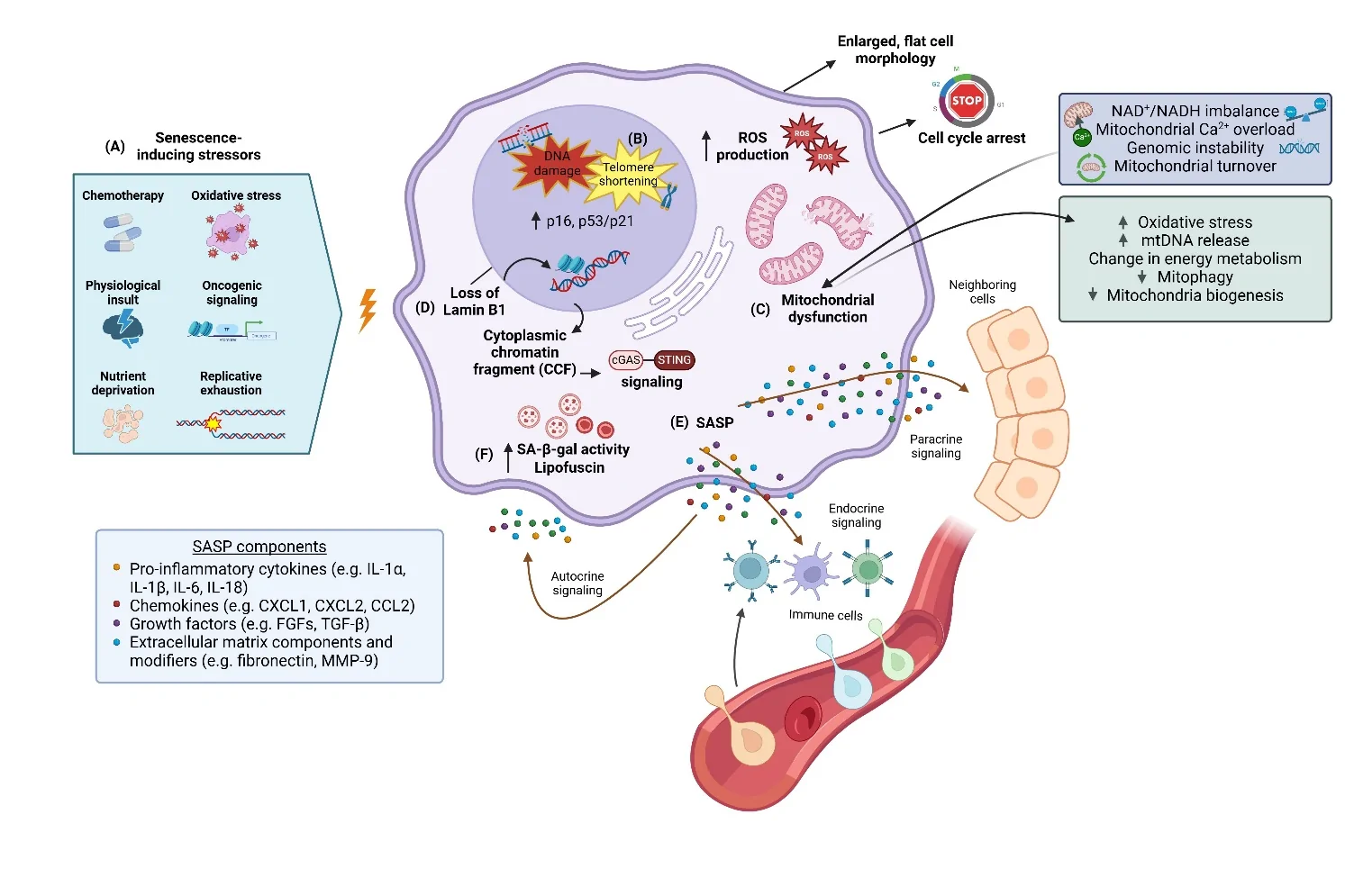
**Mechanisms of macrophage senescence**. (**A**) Exposure to senescence-inducing stressors activates the DNA damage response pathway in macrophages. This leads the cells to adopt a state characterized by cell cycle arrest while maintaining metabolic activity. (**B**) Proliferation-induced telomere shortening can also activate the DNA damage response, triggering the p16 & p53-p21 signaling cascade. These pathways repress cell-cycle regulatory genes, resulting in cell-cycle arrest and the induction of senescence. (**C**) Mitochondrial dysfunction can contribute to metabolic alterations, exacerbate oxidative stress, and promote the development of cellular senescence in macrophages. (**D**) The loss of lamin B1 leads to the release of chromatin fragments into the cytoplasm, triggering the aberrant activation of the cGAS-STING signaling pathway. This activation promotes the senescence-associated secretory phenotype (SASP). (**E**) The SASP phenotype is characterized by the secretion of an array of pro-inflammatory cytokines, chemokines, growth factors, and matrix metalloproteinases. (**F**) Senescent macrophages display high activity of senescence-associated β-galactosidase (SA-β-gal), indicating that they have entered a state of replicative senescence.

Cellular senescence is a stress response mechanism that permanently halts the cell cycle, preventing the replication of diseased, old, or damaged cells. Although senescent cells do not proliferate, they are metabolically active and are distinguished by their altered phenotype, chromatin organization, and cell morphology [74]. Senescence can be induced in younger tissues by different stressors, such as oxidative stress, physiological insults, depletion of nutrient, oncogenic stress, genotoxic stress, or replicative exhaustion (Fig. 1A). Exposure to these stressors along the lifespan cause chronic accumulation of adversity and is a recognized driver of aging that contribute to chronic low-grade inflammation. During aging, the pro-inflammatory microenvironment can exacerbate cellular senescence through oxidative and DNA damage, dysregulation of ubiquitin-proteasome system, and induction of mitochondria dysfunction [70, 75]. Senescent cells are also marked by increased levels of CDK inhibitors and changes in cellular architecture and morphology. They exhibit increased cellular volume and lysosomal activity [76]. Interestingly, senescence can be transmitted to neighbouring cells through SASP, increasing the overall senescence burden [77]. Hall et al. demonstrated that immune cells, including macrophages, can be induced to enter a senescent state by the senescent cells *in vivo* [78]. In this context, we recapitulate the major molecular and functional changes in macrophages during senescence, highlighting both specific features of macrophage senescence and common hallmarks of cellular senescence shared by senescent macrophages.

### 3.1 Hallmarks of macrophage senescence

scRNA-seq has unveiled novel molecular signatures to senescent macrophages, identifying specific markers characteristic of this cell type. Despite the distinct cellular context of macrophages compared to other cell types, senescent macrophages share some common features with other senescent cells. These include the increased expression of p16^INK4a^, senescence-associated β-galactosidase (SA-β-gal), and factors integral to the SASP.

#### 3.1.1 Specific molecular hallmarks in senescent macrophage

Senescent macrophages exhibit a variety of molecular signatures specific to their cell type. Tedder et al. identified upregulation of CD22 in aged microglia, which plays a role in regulating phagocytosis [79]. Co-expression of CD22 and TMEM119 was observed in the thalamus and cerebellum of aged mice, while these markers were mostly absent in the brains of young mice. Interestingly, inhibiting CD22 restored phagocytic function in the microglia of aged mice [69, 79]. In a study on skeleton aging, elevated levels of grancalcin (GCA) were found in macrophages. GCA expression was higher in the bone marrow supernatant and macrophages of 24-month-old rats compared to that of the 3-month-old rats. Mice with GCA knockout in macrophages showed improved skeletal aging, evidenced by an increase in osteocalcin^+^ osteoblasts, while pre-mature skeletal aging was observed in young mice applied with recombinant GCA [80]. Additionally, GLUT1 plays different roles in various tissues. The glycolysis was impaired in the macrophages of aged muscle, and GLUT1 expression was suppressed, consistent with scRNA-seq results showing disrupted inflammatory transcriptome in macrophages [81]. On the other hand, an *in vitro* study with bone marrow-derived macrophages revealed that a high level of GLUT1 transcription promoted glucose uptake [81]. Moreover, cells co-localizing GLUT1/F4-80/p16 were widespread in periodontal lesions of a diabetic mouse model, promoting an early SASP response indicated by elevated IL-1β levels. Inhibition of GLUT1 resulted in a marked reduction in mTOR phosphorylation, NF-κB and p16/p21 signaling, as well as the SASP magnitude [82]. These findings suggest that some of the molecular changes in senescent macrophages are specific to the disease or age-associated condition.

#### 3.1.2 General features in cellular senescence shared by senescent macrophages

##### 3.1.2.1 Enhanced expression of Cyclin-dependent kinase inhibitor - p16^INK4a^

The expression of p16^INK4a^ is a hallmark of cellular senescence. Research by Baker et al. demonstrated that pharmacologically eliminating p16^INK4a^-positive cells could delay the onset of age-related pathologies [83]. In a subsequent study, the same group reported that clearing p16^INK4a^ cells attenuated disease progression in established age-related conditions [84]. Focusing on macrophage senescence, Hall et al. identified a subpopulation of immunocytes that might contribute to aging. This subpopulation, which expressed macrophage surface markers and exhibited phagocytic capabilities, was recruited by senescent cells and acquired senescence-like traits, such as increased p16^INK4a^ expression and β-galactosidase activity. They also found that the number of senescent-like macrophages was higher in the tissues of old mice compared to young mice, suggesting an age-associated accumulation of senescent-like macrophages [78]. These findings align with research by Liu et al., who generated a murine reporter strain of p16^INK4a^ and characterized p16^INK4a^-fluorochrome expressing cells. They demonstrated that the frequency of p16^INK4a^-expressing cells correlated with age. Macrophages expressing p16^INK4a^ exhibited senescence characteristics such as increased SA-β-gal activity, reduced proliferation, and elevated mRNA expression of SASP-related factors [85]. Additionally, primary murine microglia cultures exhibited increased expression of p16^INK4a^, p21^CIP1/WAF1^, and p53, along with heightened SA-β-gal activity over time in the presence of telomere attrition (Fig. 1B). However, microglia isolated from the brains of aged mice only showed a modest increase in p16^INK4a^ expression and lower levels of telomere attrition [76, 86].

While removing p16^INK4a^-expressing cells can enhance health in the context of aging, some literature suggests that increased p16^INK4a^ expression in macrophages may not necessarily indicate cellular senescence. Cudejko et al. reported that p16^INK4a^ expression elevates during macrophage maturation and that macrophage differentiation’s cell cycle progression is unaffected by p16^INK4a^ levels [87]. Another study demonstrated that p16^INK4a^ promoter activity is involved in macrophage polarization towards an anti-inflammatory phenotype, independent of cell cycle regulation [88]. This aligns with findings that p16^INK4a^ expression in tumor-associated macrophages (TAMs), which exhibit an anti-inflammatory phenotype [89]. Thus, p16^INK4a^ may play various roles in macrophage physiology, and relying solely on p16^INK4a^ to identify senescent macrophages is limited. Further considerations are necessary for developing senolytic compounds targeting p16^INK4a+^ cells.

##### 3.1.2.2 Induction of senescence-associated β-galactosidase (SA-β-gal) activity

Induction of β-gal activity is commonly associated with age-related conditions due to the increased lysosomal activity and content. The lysosomal β-gal activity can be detected at pH 6.0 in senescent cells (Fig. 1F) [90]. Multiple studies have demonstrated that implanting human senescent fibroblasts into SCID mice leads to the recruitment of macrophages expressing p16^INK4a^ and β-gal [78, 85]. In a barium chloride-induced skeletal muscle injury mouse model, SA-β-gal expressing macrophage infiltrated the injury site to aid muscle regeneration. The ECM in the damaged muscle fibres of aged mice influenced the phenotype and senescence of macrophages, resulting in the expression of SASP markers. Treatment with senolytics, such as dasatinib and quercetin improved physical function [91]. In another murine model, a prodrug targeting SA-β-gal effectively eliminated senescent cells, reduced macrophage infiltration into the liver of aged mice and attenuated the inflammatory factors in both lung and liver. The authors demonstrated that targeting SA-β-gal to ablate accumulated macrophages reduced chronic inflammation and ameliorated the health state in aged organisms [92]. Nevertheless, identifying senescent cells cannot rely solely on SA-β-gal activity, as this marker is also found in quiescent primary cells or immortal cell lines under conditions of serum starvation or high confluency [90, 93].

##### 3.1.2.3 Production of senescence-associated secretory phenotype (SASP)

In addition to arresting the cell cycle, senescent cells remain metabolically active and exhibit an altered protein expression profile. One of the primary hallmarks of senescent cells is the production of bioactive pro-inflammatory secretomes, known as SASP (Fig. 1E). In aged individuals, the levels of pro-inflammatory markers are elevated 2 to 4 times compared to younger individuals [75]. While the composition of SASP varies depending on the cell type and the nature of induction, it primarily includes a group of soluble and insoluble factors such as growth factors, pro-inflammatory cytokines, chemokines, proteases and extracellular matrix components [94]. These factors modify the tissue microenvironment, interact with cell surface receptors of neighbouring cells, and activate the signaling pathways associated with age-related pathologies.

SASP is regulated by various mediators, including the inflammasome, RIG-I, pathogen-associated molecular patterns (PAMPs) or damage-associated molecular patterns (DAMPs). Inflammasome is an intracellular multiprotein complex that detects both pathological and physiological stimuli. Its activation is triggered by microbial invasion or DAMPs in innate immune cells, leading to the production of pro-inflammatory cytokines that amplify inflammation. The IL-1β and IL-18 in the SASP are able to trigger the production of pro-inflammatory factors in the surrounding cells, such as TNF-α, IFN-γ, and ROS. Macrophages are one of the major cell types where inflammasome can be potently activated, playing a significant role in aging and aging-associated CNS disorders [95, 96]. In age-related neurological diseases, the amplification of these factors through inflammasome activation can accelerate neural inflammation, impair the BBB and attract peripheral leukocytes [95, 97].

Bromodomain-containing protein 4 (BRD4) was proven to participate in the epigenetic regulation of cellular senescence and SASP, particularly in controlling the expression of inflammatory cytokine genes. In oncogene-induced senescence, BRD4 has been shown to bind to acetylated histones and is recruited to super-enhancers (SEs) adjacent to the SASP genes. The binding is essential for SASP expression and its downstream paracrine signaling [98]. Supporting this, the pharmacological inhibition or genetic deletion of BRD4 in various cell models has been shown to reduce SASP-related gene expression [98, 99]. In the lipopolysaccharide (LPS)-induced senescent macrophage model, LPS stimulation activates NF-κB signaling, which triggers BRD4 redistribution on chromosomes and leads to the expression of SASP-related genes [99, 100]. On the contrary, inhibiting BRD4 can prevent LPS-induced macrophage senescence and lipid accumulation by mitigating SASP expression in both autocrine and paracrine pathways [99]. These findings indicate that BRD4 is crucial in regulating SASP at the epigenetic level and plays a significant role in downstream autocrine and paracrine signaling.

The mechanistic target of rapamycin (mTOR) signaling also plays a significant role in the post-transcriptional regulation of cellular senescence. The mTORC1 complex enhances NF-κB activation and IL-1α translation [101, 102]. Additionally, mTOR regulates SASP via 4EBP1, which controls the translation of MAPKAPK2. MAPKAPK2, in turn, phosphorylates ZFP36L1 in senescent cells, preventing ZFP36L1 from degrading SASP-related RNA transcripts [101, 103]. A recent study by Keane et al. confirmed that mTOR complex 1 signaling and its associated translation-related genes are upregulated in the microglia of aged mice. The eradication of Rheb1, an upstream activator of mTORC1, reduced cytokine expression levels and mitigated microglial activation in these mice [104]. This effect was dependent on the phosphorylation of 4EBP1, which facilitates the binding of eIF4E to EIF4G [69, 104]. Therefore, targeting the post-transcriptional mediation of SASP-related genes presents a potential therapeutic option for age-associated disorders.

##### 3.1.2.4 Loss of nuclear fibrillar protein B1

Change in the level of nuclear fibrillar protein B1 (lamin B1) is another senescent characteristic associated with macrophages and other cell types. While an increase in lamin B1 expression level has been observed during brain cell differentiation [105], cellular senescence leads to loss of lamin B1, compromising the integrity of the nuclear envelope. This loss is followed by the migration of chromatin fragment-containing nuclear membrane to the cytoplasm, producing cytoplasmic chromatin fragments (CCF) (Fig. 1D) [106]. The CCFs are recognized by the cyclic GMP-AMP synthase (cGAS) in the cytoplasm, activating the stimulator of interferon genes (STING). The cGAS-STING signaling pathways mediate DNA damage-induced SASP and tissue inflammatory damage [106]. It has been reported that the loss of lamin B1 lead hinders neurogenesis and neuronal migration, contributing to the senescent phenotype [105].

##### 3.1.2.5 Elevated Matrix Metalloproteinase-9 level

Matrix Metalloproteinase-9 (MMP-9) is a zinc-containing endopeptidase produced by various cells, including macrophages, neutrophils, endothelial cells, fibroblasts and cardiomyocytes. Due to its ability to degrade extracellular matrix components, MMP-9 plays a crucial role in e numerous physiological processes, such as tissue remodelling and angiogenesis [107]. In CNS, MMP-9 is essential for neural synaptic plasticity and impacts learning and memory functions. Moreover, it is involved in neuroinflammation [108], regulating BBB permeability [109], and Aβ metabolism [110]. Elevated plasma levels and activity of MMP-9 have been observed in AD and mild cognitive impairment [111, 112], as well as in various models of senescent macrophages [113, 114]. Besides, MMP-9 has been linked to cardiac ageing; MMP-9 null mice exhibit biological markers indicating a younger age than their chronological age, as evidenced by reduced inflammatory markers [115]. These findings suggest that MMP-9 plays a significant role in macrophage senescence.

##### 3.1.2.6 Lipofuscin accumulation in lysosomes and cytosol

Lipofuscin, an autofluorescent lipopigment, accumulates in aging neurons, skin and muscle [90, 116]. It primarily consists of lipids, metals and misfolded proteins [90, 117]. During aging, lipofuscin accumulates in lysosomes and cytosol, leading to reduced cellular survival and diminished autophagocytotic capacity [118]. Sierra et al. observed that aged mice microglia contained lipofuscin granules, altered granularity, and increased mRNA levels of both pro-inflammatory and anti-inflammatory cytokines [119]. This finding aligns with the study by Vida et al., which demonstrated that the macrophages from aged mice had impaired functions and higher lipofuscin oxidizing compounds, as well as reduced antioxidant defences such as catalase activity and GSH levels (Fig. 1F) [120]. These data implied that lipofuscin accumulation is closely associated with cellular senescence.

### 3.2 Other alterations in senescent macrophages

In addition to the previously mentioned characteristics, aged macrophages exhibit several features commonly observed in other cell types as they age. For instance, macrophages increase in size and develop more projections, likely due to enlarged cytostomes [75, 121]. As a result of shortened telomeres and decreased telomerase activity, aged macrophages are more susceptible to oxidants and have higher intracellular levels of ROS [122, 123].

#### 3.2.1 Altered immune function in senescent macrophages

Macrophages transition into a senescent state and undergo specific functional changes in response to detrimental stimuli such as oxidative or DNA damage. As senescent cells accumulate in tissues during aging, metabolism, immune functions and polarization tendencies of macrophages gradually alter. Here, we summarize the key immune functional changes observed in senescent macrophages.

##### 3.2.1.1 Autophagy

Autophagy is an essential mechanism that regulates cellular homeostasis by degrading and removing intracellular protein aggregates and damaged organelles in the cytoplasm, recycling these components for reuse [124]. This function is indispensable for the maintenance and survival of the cells. Stranks et al. demonstrated that autophagy has an important role in preventing immunosenescence, as evidenced by a significant reduction in autophagic flux in the macrophage of aged mice compared to their younger counterparts. Deleting the essential autophagy gene, Atg7 increased the macrophage population and pro-inflammatory cytokine response while diminishing phagocytic function and nitrite burst capacity. These findings suggest that modulating autophagy may help preserve macrophage function and prevent excessive inflammation during ageing [125].

Impaired autophagy is also implicated in neurodegeneration. As microglia age, their ability to clear misfolded proteins diminishes, leading to neuroinflammatory responses in the brain [12]. In AD, the suppression of autophagy in microglia results in their disengagement from amyloid plaques, exacerbating pathology in mice. In contrast, removing senescent microglia with autophagy deficiencies ameliorates neuropathology. Autophagy deficiency leads to reduced cell growth, increased p21^Cip1^, SASP and dystrophic morphologies, indicating that microglia undergo senescence [126].

##### 3.1.2.2 Phagocytosis

Macrophages, as professional phagocytes of the innate immune system, recognize microbe-associated molecular patterns (MAMPs), DAMPs, and foreign particles *via* various pattern recognition receptors (PRRs). They internalize antigens *via* phagocytosis and initiate innate immune responses [26]. Studies have reported a decline in phagocytic ability in aged macrophages. For instance, aged macrophages in CNS showed diminished capacity to remove myelin debris due to disrupted expression of genes in the retinoid X receptor pathway. This impairment can be partially restored by retinoid X receptor agonists [127]. Moreover, primary peritoneal macrophages from young mice demonstrate a stronger ability to engulf fluorescent *E. coli* particles compared to those from aged mice [69, 128]. Another study found that *S. pneumoniae* infection induced microtubule-associated protein 1 light chain 3-associated phagocytosis, which was compromised in aged bone-marrow-derived-macrophages (BMDMs) compared to the young BMDMs [129].

##### 3.1.2.3 Antigen presentation

In contrast to younger adults, macrophages from aged individuals exhibit decreased antigen-presenting capabilities [27]. As one of the major antigen-presenting cells, macrophages constitutively express MHC class II (MHCII) molecules under steady-state conditions [130]. These molecules present antigen-derived peptides to CD4^+^ T lymphocytes, a crucial step in triggering adaptive immune responses [131]. Nevertheless, it has been reported that in aging macrophages, the expression of MHCII molecules, along with the CD80 and CD86 co-receptors, is downregulated, leading to reduced capacity for antigen presentation [132, 133]. Additionally, the expression of TLR is also compromised in aged macrophages. The mRNA expression of TLR 1-9 is reduced in aged mouse macrophages, and the protein level of TLR4 is similarly reduced [134]. Furthermore, the TLR2 and TLR4 co-receptor CD14 showed lower expression in macrophages from aged mice compared to their younger counterparts [135]. These findings indicate that reduced TLR expression may contribute to the decreased antigen-presenting capacity of aged macrophages, thereby playing a role in immune-suppression.

### 3.3 Mitochondrial dysfunction in senescent macrophages

Mitochondrial dysfunction is closely linked to age-associated inflammation due to increased energy demand for biosynthesis. Mitochondrial homeostasis is governed by mitochondrial biogenesis, mitophagy, and mitochondrial dynamics. In senescent cells, mitochondria exhibit structural changes, including increased size and volume. The increase in mitochondrial mass is associated with declined mitochondrial fission and defective mitophagy degradation [136]. Mitophagy is the mitochondrial-specific autophagy process that clears damaged mitochondria. Disrupted mitophagy is linked to a deficiency in S-nitrosoglutathione reductase, leading to an excessive S-nitrosylation of Parkin, reduced fission and impaired mitophagy [137]. Besides, maintaining mitochondrial dynamics is also important in controlling mitochondria size, shape, number and distribution, achieved via the fission and fusion processes. Mitochondrial fusion enables the exchange of intact Mitochondrial DNA (mtDNA) with the damaged mtDNA, with OPA1 and Mfn1/2 being the key fusion proteins. On the other hand, adaptors such as FIS1 and Mff facilitate the anchoring of Drp1 protein to the outer mitochondrial membrane, initiating mitochondrial fission to produce new mitochondria. Disruption of Mff-Drp1 interaction can lead to mitochondria dysfunction [138]. Accumulated oxidative damage can lower FIS1 levels, resulting in giant mitochondria, and the elongation of mitochondria is associated with the activation of senescence-associated pathways [139, 140].

Genomic instability is also a significant contributor to mitochondrial dysfunction. In contrast to nuclear DNA, mtDNA lacks the protection by introns and histone, and mitochondria do not have an effective mtDNA repair system. This makes mtDNA prone to mutations as compared with the nuclear DNA [141]. In post-mitotic tissues, these mutations tend to accumulate with age [142], and clonal expansion of point mutations from individual cells is the major driving force for this deterioration [143], particularly when mutations affect the mtDNA replication or repair mechanisms [144]. This is evidenced by the pre-mature aging phenotype observed in mitochondrial mutator mice with mtDNA deletions [145] and the cellular senescence caused by mtDNA depletion [146]. Studies have reported the amount of mtDNA mutations were elevated with a decreased mtDNA copy number in different tissues of aging organisms [10, 147, 148]. The accumulation of mtDNA mutations is physiologically relevant to premature aging [149], and the defective proofreading ability of mtDNA polymerase compromises mtDNA integrity [150]. Mitochondria, functioning as the powerhouse in cells, can experience electron leakage during respiration, leading to the formation of superoxide and oxidative stress, which further increases mtDNA mutation rates [151]. Oxidative stress can also cause mtDNA to be released into the cytoplasm [147], where it binds to cGAS and activates the STING through the messenger cyclic GMP-AMP (cGAMP) [152]. The cGAS-STING pathway is closely related to SASP and cellular senescence [153]. Furthermore, mtDNA can be exported extracellularly as circulating cell-free mtDNA, contributing to systemic inflammation and neurological disorders [154]. Neurodegeneration and advanced age are associated with reduced levels of melatonin, an endogenous free radical scavenger. Melatonin deficiency disrupts mitochondrial homeostasis, triggering mtDNA release, activating the cGAS-STING-IRF3 pathway, and leading to the secretion of pro-inflammatory cytokines [155].

The maintenance of the NAD^+^/NADH ratio is another crucial factor in controlling mitochondrial metabolism. During aging, there is a gradual decline in NAD^+^ levels, yet regulating the NAD pool is essential for many vital functions. NAD^+^ is a pivotal cofactor in ATP production and matrix metalloprotein maintenance, it also acts as a substrate in various redox reactions [144]. The balance between cellular NAD^+^ and NADH levels is modulated by their synthesis and degradation. Studies have reported that the *de novo* NAD synthesis in aged mouse peritoneal macrophages and human monocyte-derived macrophages (MDM) were decreased, along with a reduced mitochondrial sirtuin (SIRT)-3 activity. This is also accompanied by a reduction in complex I function, lowered phagocytic capacity and pro-inflammatory phenotype [156]. SIRT3 is the main deacetylase in mitochondria [157], its abolition results in mitochondrial dysfunction-associated senescence (MiDAS) and the induction of SASP. Another sirtuin protein, SIRT5, also exhibits suppression and SASP modulation functions in a lesser extent [158]. Of note, activation of SIRTs is also related to mediating inflammatory responses produced by macrophages/monocytes in acute inflammation [159]. A recent study by Zhan et al. reported that PARP-1 and SIRT3 play roles in connexin 43 (CX43) deletion-induced acceleration of BBB dysfunction during aging, which is linked to the decline in NAD^+^ levels and mitochondrial dysfunction. NAD^+^ levels could be rescued by dietary supplementation of PARP-1 pharmacologic inhibitors such as Olaparib or nicotinamide mononucleotide, alleviating BBB leakage in aged mice [160]. It is noted that other NAD^+^ consuming enzymes, such as PARPs, CD38, CD157, or SARM1, also degrade NAD. For instance, recent research has shown that dysregulated cholesterol metabolism in macrophages leads to upregulated CD38, resulted in NAD^+^ depletion and contributed to subretinal drusenoid deposits and neurodegeneration *in vivo.* The author also demonstrated that cellular senescence and macrophage dysfunction could be reversed by supplementing NAD^+^, and the administration of healthy macrophages could enhance cholesterol and senescent macrophages clearance in age-related macular degeneration [161].

Cellular calcium (Ca^2+^) homeostasis is critically regulated by mitochondrial activity, and the dysregulation of mitochondrial Ca^2+^ level is implicated in cellular senescence and age-associated conditions. When cytosolic Ca^2+^ concentration increases, mitochondria intake Ca^2+^ through voltage-dependent anion channels (VDACs) and the mitochondrial calcium uniporter (MCU) into the mitochondrial matrix. This Ca^2+^ transfer helps in preventing cytosolic Ca^2+^ overload while it may instead lead to Ca^2+^ overload in the mitochondria [162], thereby results in mitochondrial dysfunction which is characterized by reduced ATP production, dropped in membrane potential, and increased ROS production [163, 164]. On the contrary, a recent study analysing the transcriptomic profile of 700 blood samples revealed that the MCU channel and its regulatory subunit MICU1 are downregulated gradually with age [165]. The finding aligns with subsequent studies showing significant reductions in mitochondrial Ca^2+^ uptake capacity and defective phagocytosis in aged mouse macrophages. Both human and mouse macrophages demonstrated that decreased mitochondrial Ca^2+^ uptake enhances cytosolic calcium oscillations and potentiates NFAT and NF-κB activation, leading to a hyper-responsive state to inflammatory stimuli, including purinergic signals [165]. These studies highlight the link between age-associated alterations in mitochondrial physiology and macrophage-mediated inflammation, proving that the reduction in macrophage mitochondrial Ca^2+^ uptake can be a significant driving force for inflammaging. Based on these findings, it can be postulated that restoring mitochondrial Ca^2+^ uptake capacity in tissue-resident macrophages could potentially reverse inflammaging of specific organs, especially under age-associated conditions that covers various types of neurodegenerative diseases.

Mitochondria play a crucial role in inflammation [166] and inflammaging [167] as they provide the energy and essential metabolic/immune intermediates needed for proper immune cell functions, including cytokines, chemokines and prostaglandins. Prostaglandin E2 (PGE2), a lipid signaling molecule, tends to accumulate in the macrophages of aged individuals and regulates immunity. PGE2 has been identified as a suppressor of the antigen-presenting cells by controlling the antigen-presentation ability and contributing to the suppression of T cell function during aging [168]. In aged macrophages, PGE2 exhibits pro-inflammatory properties. It triggers glucose sequestration into glycogen through the AKT-GSK3β-GYS1 signaling pathway. This process results in a reduced mitochondrial respiration and ATP production, thereby affects *de novo* NAD synthesis, limiting the deacetylation of mitochondrial complex II subunit by SIRT3 and reducing the succinate dehydrogenase activity [156]. Consequently, succinate accumulates and stabilizes HIF-1α, an activator of pro-inflammatory cytokines [20]. Additionally, increased PGE2 synthesis in the peritoneal macrophages of aged mice is attributed to the upregulated NF-κB and cyclooxygenase-2 (COX-2) activities [169]. Conversely, genetic or pharmacological inhibition of PGE2 or EP2 receptor can direct peripheral macrophages and microglia towards an anti-inflammatory phenotype, decreasing pro-inflammatory cytokine production in blood and hippocampi. This shift alleviates hippocampal synaptic plasticity deficits and improves spatial memory in aging mice [20]. These findings highlight the potential of reprogramming glucose metabolism to restore cognitive function in aging.

Taken together, mitochondrial homeostasis is affected by multiple factors, such as NAD^+^/NADH ratio, calcium homeostasis, genomic stability, and mitochondrial biogenesis and dynamics. Dysfunctional mitochondria would lead to an increase in oxidative stress, released of mtDNA into the cytoplasm, change in energy metabolism, secretion of pro-inflammatory cytokines, as well as dysregulated mitophagy and mitochondria biogenesis (Fig. 1C). These consequences are closely linked to age-associated inflammation.

### 3.4 Inflammatory mediators associated with senescent macrophages

Macrophage senescence can be induced by various factors, and the SASP they secreted can amplify the pro-inflammatory signals to neighbouring cells. For instance, as mentioned above, the activated PGE2 in aged macrophages can reduce succinate dehydrogenase activity and results in the stabilization of HIF-1α [20]. Previous studies have reported elevated IL-1β, IL-6, and TNF-α levels in aged macrophage/microglia [12, 119]. A meta-analysis revealed that TNF is the most frequently reported cytokine in aging macrophages, followed by IL-6 and IL-1β [121]. Additionally, research has shown that activated microglia in PD exhibit increased ICAM-1 expression, leading to enhanced secretion of TNF-α, IL-1β, and IL-6, as well as increased nitric oxide production, which regulates the neuroinflammatory process [170, 171]. Moreover, IL-1β, a key component of the SASP, can be detected in the brain, CSF, and serum of AD patients. Functionally, IL-1β promotes the synthesis of neuron Aβ precursor protein and Aβ, as well as the phosphorylation of tau protein, leading to the formation of neurofibrillary tangles [172, 173].

Intriguingly, the cytokine profile of SASP secreted by macrophages is also influenced by the tissue environment and cell-cell interactions. During CNS inflammation, activated T cells secrete pro-inflammatory cytokines such as GM-CSF and IL-17, which activate NF-κB transcription and decrease the aryl hydrocarbon receptor (AHR) expression in the CNS-resident glial cells, including microglia. These cytokine molecules trigger microglia to secrete pro-inflammatory molecules such as IL-1α, IL-1β, C1q, TNF-α, and VEGFB while downregulating the anti-inflammatory cytokine TGF-α. These mediators in turn can crosstalk with other resident cells, such as astrocytes, and recruit encephalitogenic T cells and pro-inflammatory monocytes, thereby reinforcing CNS inflammation [31].

Furthermore, a recent study discovered that the deficiency in type 2 cytokine signaling is associated with aging phenotype. Macrophages with reduced IL-4-STAT6 signaling were more susceptible to senescence and less capable of maintaining the expression of DNA repair proteins in homologous recombination and Fanconi anemia pathways. This reduction in STAT6 expression led to the release of nuclear DNA into the cytoplasm, causing tissue inflammation. Interestingly, the study also demonstrated that the IL-4 treatment had effects comparable to senolytics, preventing macrophage senescence [174]. Additionally, inflammatory cytokines in the SASP can promote macrophage proliferation. Pro-inflammatory macrophages tend to accumulate in metabolic tissues, exhibiting high levels of CD38, which enhances CD38-dependent NADase activity and increases macrophage NAD consumption [175]. This may partially explain the decline in NAD levels in tissue with age [176]. These findings indicate that cytokine induction is a key factor in interacting with different cell types and regulates macrophage senescent status.

### 3.5 Macrophage senescence and neurodegenerative diseases

Targeting senescent myeloid cells offers a promising approach to treating neurodegenerative diseases. However, it remains unclear whether senescence affects the population of BAMs in the meninges, perivascular spaces and choroid plexus [177]. On the other hand, the characteristics of senescent microglia are better understood. Senescent microglia exhibit reduced migration and phagocytic capabilities, implying that genetic differences between homeostatic and disease-state microglia contribute to disease pathology. Moreover, the inflammasome, primarily from microglia and macrophages, is closely linked to the development of neurodegenerative disorders in aging brains. Therefore, strategically targeting mutated genes and inflammasome activation in microglia and macrophages presents a rational approach for designing preventive and therapeutic measures against age-related CNS disorders.

#### 3.5.1 Dementia

Research has shown that the expression of various inflammasome components is increased in the hippocampus of aged mice [178]. In senile microglia, the expression of the inflammasome sensor, NLRP3, was enhanced compared with the younger counterparts, leading to the activation of caspase-1 [179]. This enhancement is associated with a decline in neurological functions in aged mice, even without disease. By eradicating the NLRP3 inflammasome in aged mice, motor function and cognitive performance can be ameliorated in an IL-1-dependent manner [95, 179]. A recent study confirmed that spermidine and spermine could potentially alleviate aging-induced dementia. These compounds were shown to mediate AMPK phosphorylation, reduce the levels of NLRP3, IL-1β, and IL-18, regulate autophagy proteins, and improve mitochondrial function in senescence-accelerated mouse-8 (SAMP8) [180].

#### 3.5.2 Alzheimer’s disease

The current research on AD primarily focuses on the functional change in microglia, such as their role in clearing Aβ aggregates. Initially, dystrophic microglia morphology was thought to be associated with senescent microglia [181]. Studies have found more dystrophic microglia in AD patients than in age-matched groups [182]. A recent work by Ng et al. using a P301S mouse model demonstrated that a specific population of disease-associated microglia expressing Ccl4 differs from the non-senescent inflammatory microglia. These Ccl4^+^ exhibit high expression of senescence-associated genes and undergo cell cycle arrest [183]. The study also showed an enrichment of Ccl4^+^ microglia in the brains of tau mice. It was observed that Ccl4^+^ microglia could be both dystrophic and hypertrophic, while the dystrophic microglia can also be Ccl4^-^ [183]. This implies that, although there can be overlap between senescent microglia and those with a dystrophic morphology, the senescent state in microglia cannot be determined solely based on morphology.

In addition to morphological changes, genetic variations related to specific microglia functions, such as phagocytosis, contribute to AD pathology. Genes including TREM2, ABI3, CD33, PLCG2, and INPP5D have been identified as playing roles in the deposition and microglia phagocytosis of Aβ in the brain [184-186]. Postmortem transcriptomic data of AD patients revealed altered expression of genes related to actin cytoskeleton dynamics and cell adhesion, likely associated with the decline in cell motility [187]. Moreover, it is suggested that apolipoprotein E (APOE) produced by macroglia is pivotal for synapse maintenance [188]. APOE, essential for lipid transport [189], shows increased expression in AD and aging individuals, governing the transition of microglia from homeostasis to a neurodegenerative disease-associated state [63, 190, 191]. Different isoforms of APOE may have different functions in AD. For instance, the APOE *ε*2 haplotype is suggested to be a protective factor against AD, linked to a mitigated aging microglia phenotype [191]. Conversely, the *ε*4 isoform lacks this protective effect and likely contributes to the pathogenesis of late-onset familial and sporadic AD [192, 193]. Furthermore, inflammasome activation can result from lysosomal damage and ROS produced by microglia due to Aβ deposition in AD [173, 194]. Aβ aggregates can interact with NLRP3 and ASC, leading to inflammasome activation in infiltrated macrophages, which further promotes neuroinflammation [195]. Notably, pharmacological or genetic interference with inflammasome activation can suppress Aβ deposition and thereby ameliorate cognitive function in AD models [196, 197].

In addition to the involvement of microglia in AD, PVMΦ also exhibits characteristics of cellular senescence and contributes to disease progression. PVMΦ are involved in Aβ-induced cerebrovascular oxidative stress by upregulating the free radical-producing enzyme NOX2 [198]. Aβ affects neurovascular regulation via CD36, which activates Nox2-containing NADPH oxidase, leading to cerebrovascular oxidative stress. Depleting PVMΦ using clodronate can terminate ROS production and alleviate Aβ-induced cerebrovascular dysfunction [199]. Besides, defective neuronal Aβ clearance along the perivascular spaces, leading to cerebral amyloid angiopathy, can be improved by stimulating PVMΦ turnover, independent of astrocyte or microglia activity [200]. These findings suggest that PVMΦ could be a potential target for AD.

#### 3.5.3 Parkinson's disease

The accumulation of α-synuclein aggregates is a hallmark of PD, which can lead to mitochondrial dysfunction and neuronal death via various mechanisms [201]. Research has shown that disease-activated BAMs express genes engaged in antigen presentation, inflammation, and immune cell recruitment. The eradication of BAM subsets has been found to reduce inflammatory processes [29, 202]. Additionally, studies indicate that meningeal macrophages are involved in clearing brain α-synuclein [29, 203]. However, the role of BAM senescence in PD progression is not yet well understood.

In contrast, understanding the microglial senescence in PD is more comprehensive. Microglia is important in the clearance and degradation of misfolded α-synuclein [204]. Studies using a murine tau-dependent neurodegenerative model have revealed increased senescent microglia and astrocyte populations in PD. This finding is supported by observed improvements in cognitive function, amelioration in cortical and hippocampal neuron degeneration and lower tau hyperphosphorylation in senescent microglia/astrocytes-eradicated INK-ATTAC transgenic mice [205]. Microglia also serve as scavenger cells by their ability to degrade α-synuclein declines upon LPS stimulation, leading to cytoplasmic accumulation [204]. On the other hand, microglia are also implicated in the α-synuclein transportation in PD. For instance, the exosomes released by microglia can carry α-synuclein to neurons and induce protein aggregation [206]. Conversely, α-synuclein in exosomes can activate microglia. The transmission of α-synuclein from the plasma of PD patients via exosomes to the striatum of mice brain and the BV2 mouse microglia cell line has been demonstrated, in which the microglia exhibited a potent uptake of exosome and thereby resulted in the activation of microglia. In the BV2 cell line, the activated microglia showed elevated cytokine and nitric oxide production, dysregulated autophagy, augmented accumulation of α-synuclein and promoted release of α-synuclein into the extracellular space [207]. There is also an increased number of HLA-DR-positive cells in activated microglia in the substantia nigra of PD brains [208, 209], which may be implicated in the T-cell activation processes [191]. In addition, various types of microRNA were identified to participate in the progression of PD. The expression of miR-195 was attenuated in LPS-induced BV2 cells, and overexpressing miR-195 *in vitro* could suppress pro-inflammatory cytokine secretion while enhancing anti-inflammatory cytokine production [210]. Similarly, upregulating miR-7 or miR-30e can inhibit NLRP3 inflammasome activation in microglia, reducing pro-inflammatory cytokine induction. Administration of miR-7 or miR-30e mimics in MPTP-induced PD mouse models mitigates dopaminergic neuron degeneration and improves motor behavioural deficits [211, 212]. Furthermore, α-synuclein can induce mitochondrial ROS production and activate NLRP3 inflammasome via microglial endocytosis and the subsequent lysosomal cathepsin B release [212]. NLRP3 inhibitors can ameliorate α-synuclein aggregate accumulation and improve motor deficits and nigrostriatal dopaminergic degeneration in rodent PD models [213].

#### 3.5.4 Amyotrophic lateral sclerosis

Amyotrophic lateral sclerosis (ALS) is a neurodegenerative disease that progressively affects the motor neuron system in adults. Research using a familial ALS mouse model has demonstrated that microglia play a crucial role in triggering neuroinflammation in ALS [214]. An earlier study found that a mutation in the superoxide dismutase 1 (SOD1) gene in microglia leads to a higher TNF-α production and lower IL-6 secretion upon LPS stimulation, with these effects more pronounced with age [215]. Nikodemova et al. later reported an increased number of microglia in the spinal cord but not in the cortex of SOD1G93A rats, accompanied by reduced TNF-α and IL-6 expression at the end-stage of ALS [216]. Further evidence indicates that microglia are reactive during preclinical stages and become unresponsive at disease onset [217]. These data suggest that changes in microglial function may exist along ALS progression, and the reduced neuroprotective capabilities of dysfunctional or senescent microglia may contribute to disease progression, particularly in its later stages [218]. This is evident by another study observing dystrophic microglia in the spinal cord, brainstem, and red nucleus of SOD1G93A transgenic rats. The microglia demonstrated severe abnormalities, including cell fusion and cytorrhexis, indicating aberrant activation and degeneration [219]. Later studies showed the microglial phagocytic function is suppressed in the SOD1G93A mouse model, and these microglia displayed abnormal molecular signatures, such as loss of Tmem119, P2ry12, and Olfml3 expression [220]. Cultured SOD1G93A microglia exhibited SASP features characterized by SA-β-Gal activity, the presence of MMP-1, p16^INK4a^, p53, and nitrotyrosine [221]. These abnormalities were accompanied by the enhanced expression of miR-155, while genetically eradicating miR-155 could restore dysfunctional microglia, reverse the abnormal molecular signature, delay disease onset and prolong SOD1 mice survival [220]. Similar to other neurodegenerative disorders, NLRP3 inflammasome activation is involved in ALS development. Increased NLRP3 expression was observed in microglia from the TDP-43Q331K ALS mouse model, and the SOD1G93A activated the inflammasome in primary mouse microglia expressing NLRP3, causing cleavage of caspase-1, ASC speck formation and IL-1β secretion [222]. Notably, diphenyl diselenide inhibited NLRP3 inflammasome activation in SOD1G93A microglia by mediating the IκB/NF-κB pathway, providing neuroprotection in ALS models [223].

## 4. Therapeutic potential of targeting macrophages

Macrophages have both protective and pathogenic functions in clearing intruding pathogens and endogenous materials, including apoptotic cells, Aβ, and surfactants, and are involved in many human diseases [58, 224]. They can rapidly respond to the changes in the local microenvironment and adopt diverse transcriptional signatures, which pose a high heterogeneity in tissues [24]. The high alterability of macrophage phenotypes supports their homeostatic role in innate immunity and attracts high interest in macrophage-targeted therapy development in treating CNS diseases [225-227]. Based on the diverse functions and plasticity of macrophages in the brain, macrophage-targeted therapy provides a safe, versatile therapeutic approach that can enhance disease treatment in the brain.

### 4.1 Latest clinical trials on macrophage-targeted therapy in age-related diseases

Age-related disease is a growing concern nowadays. One promising area of research in the treatment of age-related diseases is macrophage-targeted therapy [27, 228]. Macrophages are immune cells that play a critical role in the immune response and tissue repair [229]. However, their activity can become dysregulated with age, leading to the development of age-related diseases, such as chronic inflammation and tissue damage[23]. In recent years, several clinical trials with promising results have investigated the use of macrophage-targeted therapy in age-related diseases. A clinical trial search on ‘clinicaltrial.gov’ was performed with the search term “macrophage”, and 393 search results were found. Trials on macrophage-targeted therapy in age-related diseases were manually determined and included in Table 1. Most of the current macrophage-targeted therapies focused on cancer treatment, and only 3.3% (13 out of 393) trials were relevant to age-associated diseases. Among these 13 trials, 4 trials regarding the neurodegenerative diseases are in phase 2.

The primary aim of macrophage-targeted therapies in these clinical trials is to modulate macrophage function. For instance, the leukine, which augments macrophage activity, can facilitate the clearance of amyloid deposits, potentially benefiting AD treatment by reversing associated memory impairments (registration number: NCT04902703 and NCT01409915). The suppression of TLR4/Nk-kB signaling by Pentoxifylline can also activate macrophages to enhance their function in immune response in PD (registration number: NCT05962957). Additionally, drugs regulating macrophage number, phenotype, and activity also serve as another target in treating age-related diseases (i.e. atherosclerosis) (registration number: NCT02058641, NCT02576288, NCT01258907, and NCT00695305). Although the treatment targeting macrophages achieved great progress, there are still some limitations that need to be considered. The heterogeneity of macrophages involved in targeted disease progression presents a significant issue for drug delivery specificity, raising significant concern about off-target in disease treatment. For example, leukine, a master lineage regulator of nearly all macrophages, is incapable of limiting its modulation to a specific subpopulation of macrophages that is involved in the targeted disease progression [230]. In addition, macrophages show a similar self-renewal capability as stem cells, causing drug resistance in disease treatments [231]. Targeting the macrophage self-renewal to control their population and reshaping the subpopulation pattern by modulating macrophage repolarization could serve as potential approaches in macrophage-based therapies. However, future studies are required to verify if such modulation would lead to abnormal functions in macrophages. Despite these limitations, as dysregulation of macrophage activity contributes to these diseases [232], macrophage-targeted therapy shows promise in treating age-related diseases [132, 233, 234]. Nevertheless, macrophage population changes occur in aging tissue, and targeting macrophages non-specifically can induce dysfunction and worsen disease progression[235]. Therefore, further research is needed to develop more specific therapies in targeting distinct macrophage populations to treat age-related diseases.

**Table 1:** Clinical trials of macrophage-targeted therapy for age-related diseases.

Drug	Clinical Identifier	Title	Disease	Phase of clinical trials
**Sargramostim/ Leukine/ a (GM-CSF)**	NCT04902703	Phase II Trial to Evaluate Safety and Efficacy of GM-CSF/Sargramostim in Alzheimer's Disease (SESAD)	Alzheimer Disease	Phase 2
	NCT01409915	Study of the Safety & Efficacy of Leukine in the Treatment of Alzheimer's Disease	Alzheimer Disease	Phase 2
	NCT03304821	Granulocyte-Macrophage Stimulating Factor (GM-CSF) in Peripheral Arterial Disease (GPAD-3)	Peripheral Artery Disease (PAD)	Phase 2
	NCT01041417	Granulocyte-Macrophage Stimulating Factor in the Treatment of Peripheral Arterial Disease (GPAD-2)	Peripheral Artery Disease (PAD)	Phase 2
**Intranasal auto-M2-BFs**	NCT02957123	Intranasal Inhalations of Bioactive Factors Produced by M2 Macrophages in Patients With Organic Brain Syndrome	Organic Brain Syndrome	Phase 1 Phase 2
**Pentoxifylline**	NCT05962957	Pentoxifylline and Parkinsonism	Parkinson Disease	Phase 2
**Victoza**	NCT02650206	Effect of Liraglutide (Victoza) on Inflammation in Human Adipose Tissue and Blood	Diabetes Mellitus, Type II	Phase 1
**Darapladib**	NCT02058641	Effect of Darapladib on Cantharidin-Induced Inflammatory Blisters in Subjects With Type 2 Diabetes Mellitus (T2DM)	Atherosclerosis	Phase 1
**Sitagliptin**	NCT02576288	Sitagliptin Effects on Arterial Vasculature and Inflammation in Obesity (SAVORO)	Atherosclerosis	Phase 2
**MLDL1278A**	NCT01258907	A Study to Evaluate the Safety, Tolerability, and Activity of Intravenous MLDL1278A in Patients on Standard-of-Care Therapy for Stable Atherosclerotic Cardiovascular Disease (GLACIER)	Atherosclerosis	Phase 2
**Rilapladib**	NCT00695305	An Imaging Study in Patients With Atherosclerosis Taking Rilapladib or Placebo for 12 Weeks	Atherosclerosis	Phase 2
**68GaNOTA-Anti-MMR-VHH2**	NCT04758650	Study of 68GaNOTA-Anti-MMR-VHH2 in Oncological Lesions, Cardiovascular Atherosclerosis, Syndrome With Abnormal Immune Activation and sarcoïdosis (MITRAS)	Oncological Lesions Cardiovascular Atherosclerosis Syndrome With Abnormal Immune Activation and sarcoïdosis	Phase 2
**Allocetra**	NCT06459063	Intra-articular Allocetra in Osteoarthritis of the 1st Carpo-metacarpal Joint	Osteoarthritis	Phase 1 Phase 2

### 4.2 Targeting macrophages via micro-based drug delivery

Aging is described as the primary risk factor for most neurodegenerative disorders, including AD and PD [236]. While various drugs have been developed to treat brain diseases, the main challenge in treating neurodegenerative diseases is the low drug distribution efficiency, which results from the obstruction of the BBB [237]. Recent research has shown that chemokines released from the CNS parenchyma can stimulate macrophage migration to the brain [238]. This infiltration of macrophages across the BBB provides a promising approach for improving drug delivery efficiency in the brain. The immunotherapy targeting non-parenchymal macrophages dramatically reshaped the neurodegenerative disease treatments [52]. However, developing disease-modifying drugs targeting macrophages in neurodegenerative disease treatment is still pending for investigation. Based on the phagocytosis capability of macrophages in the clearance of cellular debris, pathogens, and substances, the micro-based drug delivery system for targeting macrophages has been established in CNS disease treatments [5, 239]. It can be classified into two main categories: 1) ligand-based drug delivery systems and 2) extracellular vesicle (EV)-based drug delivery systems. By performing micro-based drug delivery, it is possible to specifically target macrophages in the brain to improve drug delivery efficiency and sustain the drug release at targeted regions, potentially leading to more effective treatments for brain diseases.

#### 4.2.1 Ligand-based drug delivery

Ligand-based systems utilize ligands that recognize receptors on the surface of different macrophage subtypes, including CD206, SR-A, Siglec-1, and CD44 in inflammatory and tumor-associated macrophages [240-245], p32 and Stabilin-2 in anti-inflammatory macrophages in artherosclerosis [246, 247], and CD163 in anti-inflammatory macrophages in inflammatory and malignant diseases [248], allowing for targeted drug delivery to specific regions to suppress disease progression [5, 249]. The common ligands decorated on nanoparticles include carbohydrates, polypeptides, antibody antigens, nucleic acids, lipids, and vitamins. Below, we will discuss the specifics of the recognition between these ligands and their corresponding receptors.

##### 4.2.1.1 Carbohydrates

The monosaccharides and polysaccharides are the two main carbohydrate-based ligands in targeting macrophages [250]. They offer advantages of low cost, high affinity and water solubility, and diversity of molecular structures [251]. Mannose is commonly used as the monosaccharide ligand in targeting the mannose receptor CD206 on macrophage surfaces [240]. It has been used as the first FDA-approved lymphatic mapping agent conjugated with lymphoseek [252]. Instead of being used as imaging agents, recently, mannose-decorated nanoparticles have been conducted for drug delivery and the delivery of biomacromolecules such as nucleic acids. For instance, Ganbold and Baigude designed the mannose-decorated curdlan nanoparticles for siRNA delivery to macrophages [253]. The mannose-modified magnetic drug-loaded liposomes also serve for idiopathic pulmonary fibrosis treatment by targeting activated macrophages [254]. Besides, the mannose derivative and residues can also target CD206 expressed on macrophages, specifically [5, 255, 256]. Incorporating the sialic acid on drug-loaded nanoparticles, which is a 9-carbon monosaccharide derivative, can also specifically bind to the Siglec-1 overexpressed on activated macrophages and showed high accumulation in inflammatory regions [244]. This application has been well explored in anti-tumor therapies, indicating a high potential in treating CNS-related diseases. Galactose is another monosaccharide sugar that shows high specificity in targeting macrophages. While the galactose demonstrated enhanced target efficiency to galactose-specific C-type lectin expressed on activated macrophages, mannose-modified nanoparticles still exhibit higher internalization by macrophages compared to galactose [250, 257].

In addition to monosaccharides, polysaccharides can also be applied on the nanoparticle surface to target different receptors expressed on the macrophages, such as TLR4, TLR2, or CD44 [258, 259]. Compared to monosaccharides, polysaccharides are long-chain macromolecules and have large molecular weights. They cannot be coated on drug-loaded nanoparticles as monosaccharides; instead, a variety of approaches have been proposed to construct nanoparticles by applying polysaccharides as the structural basis [260]. For example, Dextran, an exopolysaccharide extracted from bacteria, performs high biodegradability and biocompatibility when applied as a nanocarrier in drug delivery [261]. Miao et al. utilized the β-Dextran drug-loaded nanocarrier to target the SR-A expressed on macrophages in the intestine, and the modified macrophages can then be transported to the circulatory system and cross the BBB [262]. Instead of attempting to cross the BBB to reach parenchymal microglial, targeting tissue-resident macrophages is an effective therapeutic approach for treating CNS diseases via bypassing the barrier based on the infiltration ability of macrophages. Hyaluronic acid (HA)-based nanoparticles have been developed to act as nanomedicine for delivering drugs to the pro-inflammatory macrophages via the cell surface HA receptors (e.g. CD44) [263]. Incorporating poly(lactic-co-glycolic acid) with HA nanoparticles, these nanoparticles can be intravascular injected and were found to aggregate in hippocampi in the aged APP/PS1 AD mouse model [264]. The biofunctionalized nanoparticles hold great promise as a means of delivering therapeutic drugs for treating CNS-related diseases in aging.

Applying carbohydrate-based nanoparticles, especially mannose and dextran, increasingly enhances the targeting specificity of macrophages [250]. However, carbohydrates can be recognized by not only macrophage lectin but also a range of lectins from other cell types, like epithelial or dendritic cells [265]. For example, the mannose residues can also target the C-type lectin receptor DC-SIGN expressed on both macrophages and dendritic cells [266, 267]. Despite the high solubility and affinity, the carbohydrate-based nanoparticle can provide, the specificity targeting macrophages still needs to be improved.

##### 4.2.1.2 Antibody antigen

The antibody-antigen plays two roles in macrophage-targeted therapies - targeting ligands and macrophage activity regulators [5, 268]. Modifying the antibody onto the surface of drug-loaded nanoparticles can recognize and target macrophages specifically, which could help improve the drug delivery efficiency [269]. For example, conjugating the anti-Siglec-1 antibody onto polyethylene glycol-poly(lactic-co-glycolic acid) nanoparticles can enhance the efficacy of macrophage targeting by precisely targeting tissue-resident macrophages with Siglec-1 overexpression, which is associated with several diseases such as inflammatory and autoimmune diseases [270]. In addition to their role as target ligands, antibodies can also interact with the surface receptors on macrophages and control their activity and functions, such as phagocytosis, cytokine secretion, and immune responses [271]. A recent study led by Luo proposed the use of an anti-CD47 antibody conjugated nanoparticle to target the receptor on macrophage surfaces and regulate the phagocytic signaling CD47-signal regulatory protein (SIRP)-α, where the blockade of CD47-SIRPα interaction can promote the phagocytotic activity of macrophages. The study reported significant success in modulating macrophage phagocytosis and reducing plaque accumulation in Apoe^-/-^ mice. Notably, the anti-CD47-conjugated nanoparticle offers high specificity without inducing associated toxicity, as demonstrated in their *in vivo* study. Moreover, incorporating an NLRP3 inhibitor to control the intracellular inflammation response of macrophages presents a promising means of modulating macrophage activities from both intracellular and extracellular levels, providing a more precise and inoffensive regulatory approach for targeted drug delivery. However, a small reduction in low-density lipoprotein, cholesterol, and triglyceride is also observed and future studies are warranted [272]. Although antibodies have shown promise in targeting specific antigens, their potential for toxicity remains a significant hurdle for clinical translation. To optimize the use of antibody-based nanoparticles in macrophage-targeted therapies, it is imperative to apply rational design and efficient biosafety assessments to minimize their toxicity in disease treatments, which will facilitate the successful translation of these promising therapeutic approaches into clinical practice. Developing safe and effective antibody-based nanoparticles will enhance their therapeutic potential and improve the efficacy of drug delivery in macrophage-targeted disease treatments. Therefore, further research is necessary to advance the development of antibody-based nanoparticles and promote their implementation in clinical settings.

##### 4.2.1.3 Peptide ligands

Peptides demonstrate a more specific target to macrophages than carbohydrate-based ligands, and they provide advantages of smaller size and lower cost compared to antibody antigens [273, 274]. The peptide sequence can be determined from 1) random amino acid compositions followed by peptide library screening or 2) a structure-based design based on the bioactive sequences isolated from the natural proteins [275-277], which have a high expression on the surface of macrophages. It is reported that the peptide targeting the tyrosine kinase receptor B can be modified onto the surface of chitosan nanoparticles and showed an enhanced targeting efficacy to RAW 264.7 macrophages compared to the pure nanoparticles [278]. This peptide-functionalized nanocarrier performs a more specific and sensitive role than carbohydrate-based nanoparticles in targeting macrophages. Moreover, the gold nanoparticles decorated with peptide arginine-glycine-aspartic acid can target macrophages specifically and promote the macrophage polarization from the pro-inflammatory phenotype into the anti-inflammatory phenotype to enhance the phagocytosis of macrophages [279]. Meanwhile, Cieslewicz et al. discovered a unique peptide sequence, M2 macrophage-targeting peptide (M2pep), binding to anti-inflammatory macrophages instead of pro-inflammatory macrophages [280]. This peptide has been integrated into various types of nanoparticles. For instance, Wang et al. developed M2pep-decorated superparamagnetic iron oxide nanoparticles to enhance the M2 macrophage-targeting for improving MR imaging [281], and the M2pep-ferritin nanoparticles encapsulating CpG oligodeoxynucleotides help repolarize anti-inflammatory macrophages into pro-inflammatory macrophages and trigger the inflammatory response, serving as a promising anti-cancer therapy [282]. The modification with peptides makes nanoparticles perform sophisticated roles in disease treatment - not only as a targeted drug carrier, but also a regulator of phagocytosis by controlling the macrophage polarization. Additionally, peptides with distinct sequences can bind to the same receptor on the macrophage surface. Peptides 37pA and R4F are derived from apolipoprotein A-1 and can target the same receptor-scavenger receptor B1[283-285]. The integration of peptides and therapeutic agents has been demonstrated to improve the targeting efficacy towards cells of interest, offering a finely controlled approach to drug delivery by regulating the combination of peptides and drugs loaded into nanocarriers. Nonetheless, drug delivery systems functionalized with peptides still encounter obstacles such as limited binding affinity compared to antibodies and heightened susceptibility to protein degradation [286].

##### 4.2.1.4 Nucleic acid

The nucleic acid aptamers are oligonucleotides selected *in vitro* that exhibit high specificity in recognizing and targeting molecules of interest [287]. Their high tunability, low cost, good biocompatibility, and biological stability make these multifunctional oligonucleotides ideal for nanoparticle functionalization for therapeutic drug delivery [286, 288]. Tetrahedral framework nucleic acids (tFNA), consisting of four pieces of single-strand DNA with complementary sections to each other strand, showed excellent performance in drug carry and delivery, especially for small-molecule drugs [289]. The tFNA-microRNA-155 nanoparticle was designed by Qin et al. to direct the polarization of macrophages towards a pro-inflammatory phenotype by targeting C/EBPβ for the treatment of choroidal neovascularization-related diseases and neovascular age-related macular degeneration [289]. Using tFNA helps stabilize the drug-loaded nanocarrier *in vivo* and enhances drug delivery efficiency. In addition, Sylvestre et al. discovered an aptamer with a high affinity to anti-inflammatory macrophages. However, this aptamer can also bind to monocytes, and future work is needed to improve target specificity [290].

Nucleic acid aptamer-decorated drug delivery systems offer several advantages compared to antibody-based drug nanocarriers, such as smaller size and lower immunogenicity. However, despite these benefits, the clinical translation of nucleic acid-based nanoparticles has been hindered due to *in vivo* off-target effects and potential safety issues [291]. Moreover, these nanoparticles must resist nuclease degradation to reach the target site [292]. It is essential to address these limitations before clinical translation.

##### 4.2.1.5 Lipid and vitamins

Lipid-based nanoparticles are self-assembling spherical vesicles that consist of one or more phospholipid bilayers and can be internalized by cells due to their resemblance to cell membranes. These nanoparticles are less toxic than conventional drug delivery approaches and can transport both hydrophilic and hydrophobic compounds based on their amphipathic nature [293]. By functionalizing the nanoparticle surface with ligands, they can selectively target macrophages and control drug release, providing a more precise and effective option for treating brain diseases [294]. For example, incorporating the lipid ligand phosphatidylserine into drug delivery systems can offer a specific target for macrophages to identify and phagocytize [295], which can be useful for selective drug delivery to macrophages in treating CNS diseases.

In addition to lipid-based nanoparticles, vitamin-conjugated nanoparticles have been identified as potential candidates for targeting macrophages. The folate receptor (FR) exhibits a high affinity for the water-soluble vitamin - folic acid [296]. Functionalization of methotrexate encapsulated in polyamidoamine dendrimer with folic acid has been demonstrated to significantly enhance uptake by RAW 264.7 macrophages and macrophages isolated from the peritoneal cavity of mice [297]. As the folate receptor-β is selectively overexpressed in synovial macrophages of patients with rheumatoid arthritis [298], the use of folic acid-decorated methotrexate-loaded nanoparticles presents a promising therapeutic approach for treating rheumatoid arthritis. Moreover, Kumar et al. developed folate-conjugated chitosan-glycol nanoparticles loaded with methotrexate to treat the Wistar rat model with adjuvant-induced arthritis. The nanoparticles demonstrated excellent stability in serum following intravenous injection, highlighting the high stability of drug delivery using folate-functionalized nanocarriers and indicating their potential for targeted drug delivery applications [299].

While the nanomedicine with the introduced ligand modification has shown promising results in preclinical studies, these ligand-based nanoparticles are still in the laboratory phase [300]. Although their high flexibility and tunability provide opportunities for improving drug delivery, they also emphasize the importance of careful design and optimization. Furthermore, the potential for modified drug-loaded nanocarriers to cause abnormal changes in the activity or immune response of macrophages remains a concern. Therefore, developing efficient techniques for nanoparticle synthesis and biotoxicity verification is crucial for the future application of nanomedicine in clinical treatments.

#### 4.2.2 EV-based drug delivery

Apart from ligand-based delivery systems, the EV-based drug delivery system leverages the unique feature of EVs as the primary chemical messengers of intercellular communication [301]. This feature can be used to enhance the selective uptake of nanoparticles by macrophages and improve the efficiency of drug delivery to targeted macrophages [302]. EVs are plasma membrane-derived vesicles in the extracellular microenvironment released by macrophages. The bioactive substances they carry, such as nucleic acid, lipid, or protein, can be transferred into recipient cells through membrane fusion to affect their biological functions [303]. It is important to note that macrophage-secreted EVs vary in surface receptors and contents depending on the macrophage phenotype. In a study by Dechantsreiter et al., the protein expression in EVs was dependent on macrophage phenotype. Specifically, MHC class II cell surface receptor-human leukocyte antigen-DR (HLA-DR) was found to be expressed on 80% of EVs released by pre-activated and anti-inflammatory macrophages differentiated from human monocyte-derived macrophages, while only 40% of EVs secreted from pre-inflammatory macrophages expressed HLA-DR [304]. Furthermore, pro-inflammatory macrophages exhibited markedly elevated levels of pro-inflammatory lipids, including tetranor-12-HETE and 8-HETE/12-HETE, while these lipids were undetectable in anti-inflammatory macrophages [305]. Moreover, EVs originating from different phenotypes of macrophages exhibited contrasting regulatory effects on macrophage polarization. EVs secreted by anti-inflammatory macrophages were found to target pre-inflammatory macrophages and suppress the release of pro-inflammatory cytokines, thereby promoting macrophage reprogramming into an anti-inflammatory phenotype [306, 307]. Wu et al. introduced the hexyl 5-aminolevulinate hydrochloride into the EVs derived from anti-inflammatory macrophages, and these engineered EVs elicited anti-inflammatory effects and induced the polarization of pre-inflammatory macrophages towards an anti-inflammatory phenotype [308]. These findings hold promise for treating inflammatory diseases, including rheumatoid arthritis and atherosclerosis. The distinct surface receptors and contents of EVs and their regulatory role in macrophage phenotype switch offer a valuable opportunity for developing effective EV-based treatments for different pathologies. The selection of macrophage phenotypes is crucial for EV preparation in the targeted therapeutic intervention.

Notwithstanding, EV-based nanocarriers are constrained by their limited drug-carrying capacity [309]. Encapsulating nanoparticles in EVs can overcome such limitations while also providing an escape from immune clearance and enhancing the biocompatibility of these drug-loaded nanoparticles [5, 310]. In addition, the conjugation of ligands, such as peptides or nucleic acid aptamer, onto the EV-coated nanoparticles can further enhance their target efficacy and intensify their interaction with target cells [311]. As demonstrated by Li et al., coating the doxorubicin-loaded PLGA nanoparticles with EV membrane derived from macrophages can prevent immune clearance, and the further decoration of c-Met peptide, which is highly expressed in the triple-negative breast cancer cells, improved the targeting specificity in breast cancer treatments [312].

Macrophage-targeted therapy holds significant potential as an alternative approach for reversing the underlying pathological damage at the site of inflammation, highlighting the need for targeted nanomedicines that can safely and efficiently deliver therapeutic drugs to macrophages in the inflammatory microenvironment. Furthermore, taking advantage of macrophage infiltration into the brain, micro-based drug delivery systems targeting non-parenchymal macrophages offer a promising approach for overcoming drug delivery challenges in treating CNS diseases. Despite significant progress in developing micro-based macrophage-targeted drug delivery systems, gaps remain between the bench and clinical applications. Although the micro-based macrophage-targeted drug delivery system has been extensively used, it is important to note that nanoparticles can induce macrophage polarization and alter their cytokine profiling [313, 314]. These nanomedicines can also elicit potential immune responses that can reduce their effectiveness and lead to adverse impacts [315]. In addition, the heterogeneity of macrophages poses a challenge for micro-based drug carriers to target effectively. Moreover, the low yield and high cost of preparing these micro-based drug carriers make it difficult to scale up their usage. Nevertheless, nanomedicines can offer customized treatments for patients under different conditions. The flexibility and tunability of nanoparticle design enable its complex functions in therapeutic intervention. As such, a systematic high-throughput testing approach is required to facilitate the risk assessment of customized nanoparticles and promote their use in clinical settings.

### 4.3 Macrophage immunomodulation

Macrophages, as innate immune cells that play a pivotal role in CNS regeneration, have been demonstrated to be associated with the age-related decline of the immune system and inflammaging, and numerous studies have reported on the alterations that occur in macrophages during the ageing process [121]. It has been established that macrophage activation and infiltration in tissues can give rise to various inflammatory or autoimmune disorders, with a particular emphasis on their role in the inflammatory response associated with neurological disorders [316, 317]. Consequently, several treatments targeting macrophages have been developed for CNS-related diseases. Here, we will discuss current immunotherapies that target macrophages for treating CNS-related diseases, including macrophage depletion, re-polarization, and inhibition of macrophage infiltration.

#### 4.3.1 Macrophage depletion

Over the last few years, macrophage depletion has been identified as a potential therapeutic intervention for CNS-related diseases. Studies have shown that the elimination of pro-inflammatory macrophages can reduce the invasion of activated T cells into the brain, which in turn slows down the neurodegeneration progression [318]. This has led to the emergence of macrophage depletion as a promising therapeutic strategy for suppressing CNS-related diseases.

In AD mouse models, clodronate, which can induce macrophage apoptosis, has been applied to deplete perivascular macrophages, leading to a reduction in vascular Aβ deposition and reversing cerebrovascular dysfunction [199]. Additionally, after subarachnoid hemorrhage, the clearance of macrophages in subdural meninges and perivascular space by clodronate liposomes injection reduced neuronal cell death, perivascular inflammation, and microthrombosis [319]. Furthermore, the CSF-1R-specific kinase inhibitor PLX5622 has been orally administered in aging mice to target macrophages, resulting in a 70% reduction in macrophage numbers in mice. This depletion of macrophages led to improved nerve structure, demonstrating for the first time that macrophages are the driving force behind age-related degenerative changes [320]. In murine models, macrophage depletion by PLX5622 has also been demonstrated to alleviate CNS damage and reduce clinical signs of autoimmune encephalomyelitis by suppressing the induction of myelin-specific T cells [321]. However, while macrophage depletion has been demonstrated to be a promising therapeutic approach for arresting the progression of CNS diseases, it alone is not sufficient to promote brain recovery. Therefore, future investigations are needed to enhance the recovery efficiency after CNS damage.

#### 4.3.2 Macrophage reprogramming

Apart from macrophage depletion, repolarizing macrophage subtypes has been identified as another promising approach in CNS treatment [322]. Even though it is challenging to repair or regenerate damaged brain tissue currently, many drugs have been developed for alleviating disease-related symptoms by regulating macrophage polarization to suppress neuroinflammation [323, 324]. During neuroinflammation, macrophages initially exhibit pro-inflammatory phenotypes and secrete pro-inflammatory cytokines such as TNF-α, IL-1β, and IL-12 to sustain inflammation [325]. However, prolonged secretion of pro-inflammatory cytokines can cause tissue damage [326]. Macrophages shifting into anti-inflammatory phenotypes can release anti-inflammatory cytokines, including TGF-β and IL-10, which contribute to inflammation suppression and neurovascular remodeling, thereby helping maintain tissue homeostasis [325, 327, 328]. However, the macrophage phenotype imbalance often occurs in disease conditions, with more pro-inflammatory and fewer anti-inflammatory macrophages compared to a healthy state [15]. Targeting pro-inflammatory macrophages and shifting them into anti-inflammatory macrophages could be a potential strategy for treating CNS diseases.

Macrophage polarization involves several significant signaling pathways, such as TLR4, CSF-1R, NF-κB, and TNF-α signaling cascades [201, 329], and blocking key components in these signaling serves as a way of regulating the macrophage phenotype [323]. For instance, NF-κB is a crucial transcription factor linked to the activation of pro-inflammatory microglia. The significant silencing in NF-κB p65 by RNAi inhibits the inflammatory-related gene expression and drives the microglia polarization towards anti-inflammatory phenotypes, reducing inflammation and promoting poststroke recovery [330]. Meanwhile, recent studies have also identified therapeutic drugs for microglial repolarization by NF-κB-related signaling inhibition, including icaritin [331], analgecine [332], and shikonin [333]. They have shown great potential in microglial repolarization in CNS disease treatment. However, it is important to acknowledge that regulating macrophage polarization is complicated. Rapamycin, a mTORC1 inhibitor, has been employed to enhance the expression of anti-inflammatory macrophage markers and facilitate the shift towards an anti-inflammatory phenotype [334]. Meanwhile, the role of mTORC1 in macrophage repolarization is complex and contradictory. Inhibiting the expression of AKT kinase, which positively modulates mTORC1, shifts macrophages towards pro-inflammatory phenotypes rather than the anti-inflammatory phenotype as expected [335]. Further investigation is required to comprehensively understand and unravel the mechanisms involved in macrophage repolarization and its potential implications in disease treatments. Meanwhile, macrophages perform multifunctional roles in different tissues, and the repolarization direction of macrophage phenotypes varies among tissue regions and disease conditions [336]. For example, the anti-inflammatory macrophages also drive fibrosis in the liver and prevent regression; therefore, repolarizing anti-inflammatory macrophages towards pro-inflammatory phenotypes is required for attenuating liver fibrosis [337]. Hence, it is essential to deliver phenotype repolarization drugs specifically to macrophages within the region of interest. Another challenge is the stability and safety issue of re-educated macrophages. Since macrophages are plastic and interact with the specific tissue microenvironment and neighbouring cells, including other macrophage subtypes and astrocytes, the repolarization of these re-educated macrophages should be carefully considered.

#### 4.3.3 Macrophage migration/infiltration

Macrophage infiltration is known to be associated with chronic tissue damage [338]. In murine models, macrophage infiltration has been shown to correlate with brain atrophy, impaired neuronal function, and decreased regenerative responses [239]. In response to ischemic stroke, CD163^+^ brain-associated macrophages were found to migrate and accumulate in the ischemic region, where they also adopt pro-inflammatory phenotypes in the ischemic rat parenchyma [339], followed by the repopulation or regeneration of the damaged tissue. Meanwhile, the failure in regeneration during aging may be caused by poor macrophage recruitment in aged tissue, where macrophages show limited ability in migration and infiltration [340]. Therapeutic drugs, such as R162 inhibiting glutamate dehydrogenase-1, have been developed to improve the infiltration of aged macrophages, which has been shown to enhance functional recovery and tissue regeneration in aging [341]. On the contrary, the depletion of infiltrating phagocytes in experimental autoimmune encephalomyelitis also protected mice from axonal damage [342]. Therapeutic drugs targeting macrophage infiltration in treating CNS disease perform as a double-edged sword that should be carefully designed and validated before clinical use.

Macrophage-targeted immunotherapies have shown potential in treating CNS diseases; however, there are still limitations that need to be addressed. One of the main challenges is the non-specific targeting of macrophages, which can result in low treatment efficiency and potential side effects in other regions or organs [343, 344]. This lack of specificity can also lead to unintended consequences, as macrophages have versatile functions essential in immune responses [27]. Over-regulating macrophage phenotype may also induce other health issues. Additionally, the low yield of drugs that specifically target macrophages can result in high costs and limited accessibility for patients. Further research is needed to optimize the specificity, safety, and cost-effectiveness of macrophage-targeted immunotherapies for treating CNS diseases.

### 4.4 Mitochondrial transfer to CNS-residing cells

Mitochondrial dysfunction occurs in neurodegenerative disease progression, leading to altered calcium homeostasis, impaired energetic balance, and increased oxidative stress and causing ranges of defects in molecular and cellular levels [345, 346]. Intriguingly, mitochondria have been shown to translocate into injured axons and support axon regeneration, while the injured axons with low mitochondrial density demonstrated poor regeneration [347]. Instead of using the small molecule to target macrophages for disease treatments, it is shown that mitochondria can be transferred to the CNS-residing cells and serve as a promising potential therapeutic solution for CNS-related diseases [346]. Restoring the functionality of mitochondria by transferring the healthy mitochondria to replace the damaged unit helps enhance the metabolism and function of recipient cells.

Mitochondria can be delivered to different cell types in CNS, including neurons, immune cells, and tumor cells, through pathways such as tunnelling nanotubes, receptor cell endocytosis, extracellular vesicles, intercellular contact, and gap junction channels [348]. Recently, many studies have demonstrated mitochondrial transplantation in animal models and show great improvement in treating neurodegenerative diseases. For instance, Chang et al. injected the artificial mitochondria into the medial forebrain bundle of a rat model with PD, and neurons internalized these mitochondria, and improved mitochondrial function and reduced dopaminergic neuron loss were observed [349]. While various cell types are involved in intracellular mitochondrial transfer within the CNS region, most mitochondrial transfer-based therapies target neurons to restore their functionality [350]. Given the high plasticity and multifunctionality of macrophages in CNS disease progression, mitochondrial dysfunction in macrophages has gained high attention as a potential therapeutic target. Játiva et al. performed mitochondrial transplantation to cholesterol-loaded macrophages RAW264.7, mimicking the macrophage cell condition under atherosclerotic lesions [351]. This transplantation improves macrophage phagocytosis and reduces lipid accumulation, suggesting a potential therapeutic approach not only for restoring macrophage function in atherosclerosis but also for other CNS-related diseases.

Instead of invasive delivery methods like Intracerebral injection, intranasal delivery and intravenous administration can also deliver the mitochondria to the brain. However, the target specificity remains challenged [352]. Meanwhile, another study reported that macrophages secrete the mitochondria-containing vesicles expressed CD200R, which can be recognized by the non-canonical CD200R-ligand iSec1 on sensory neurons for pain relief [353], suggesting a potential way of directing mitochondrial delivery through micro-based decoration of mitochondrial surfaces. Xu et al. designed an engineered mitochondria compounding conjugating with cations-cysteine-alanine-glutamine-lysine peptide sequence to target macrophages within the spinal cord injury region [354]. This conjugated peptide sequence helps the recognition of macrophages in the spinal cord injury regions, and the transplantation of mitochondria ameliorates mitochondrial dysfunction in disease-associated macrophages and enhances macrophage phagocytosis of myelin debris, promoting the tissue regeneration and function recovery in mice with spinal cord injury. In addition, Liu et al. intravenously delivered mitochondria, functionalized with dextran and triphenylphosphonium, to target pro-inflammatory macrophages in Apoe^-/-^ mice, where this treatment attenuated inflammatory responses and reduced the plague burden [355]. With a combination of micro-based drug delivery systems, mitochondria derived from specific cell sources could also be delivered *via* either intracerebroventricular injection or intravenous administration to target the macrophages in the region of interest.

Mitochondrial transfer is a promising therapeutic solution for restoring cell function in disease treatments. However, mitochondrial transferring systems remain some concerns before clinical use. Firstly, it is shown that the transplantation of mitochondria cannot contribute to long-term functional neuroprotection [356]. It is unclear if the treatments can be repeatable in humans and how long this beneficial effect can last in patients. In addition, the mitochondria derived from different cell sources, such as astrocytes and mesenchymal stem cells, demonstrate different regulation of recipient cells [357-360]. Selecting the cell sources for generating mitochondria for mitochondrial transfer is essential. Moreover, mitochondrial transfer introduces foreign DNA into the recipient cell, raising an ethical concern that the foreign genome could potentially disrupt the nuclear genome and lead to mitochondrial genetic drift and dysfunction [361]. Lastly, mitochondrial transplantation may also interrupt mitochondrial homeostasis, where the interruption may lead to both beneficial and detrimental effects on recipient cells [362]. It is crucial to consider the possible toxic side effects that this intervention may induce.

### 4.5 Targeting senescent macrophages in neurodegenerative and other age-associated diseases

Macrophage senescence happens in neurodegenerative diseases and has demonstrated a strong association with aging [363]. The altered function in senescent macrophages, such as damaged metabolism, changed polarization tendency, delayed infiltration, and reduced phagocytosis, contributes to the disease progression in aged people [69]. In contrast, the elimination of senescent macrophages delayed atherosclerosis in mouse models [364]. Therefore, targeting macrophage senescence could be a promising and effective method for disease treatments. Currently, the main therapeutic agents for targeting senescent macrophages in treating aging and chronic diseases include senolytics and senomorphics [365].

Senolytics, such as quercetin and dasatinib, were first introduced in 2015 and used for senescent cell depletion [366]. The commonly used senolytics agents can be classified into several classes, including natural compounds targeting signaling like PI3K/Akt, NF-κB, Nrf2, and sodium-potassium pump-dependent apoptosis (e.g. fisetin, ouabain, and piperlongumine), protein tyrosine kinase inhibitors (e.g. dasatinib), Bcl-2 family inhibitors (e.g. A1331852, UBX1325), Bcl-2 homology 3 mimetics (e.g. navitoclax, ABT-737), MDM2/p53 interaction inhibitors (e.g. pipeline, P5091), Hsp90 inhibitors (e.g. geldanamycin, tanespimycin, alvespimycin), p53 binding inhibitors (e.g. FOXO4-DRI), histone deacetylase inhibitors (e.g. Panobinostat), mitochondria-targeting senolytics, and immune-mediated clearance by CAR T cells or vaccines [365, 367]. Several senolytics have entered clinical trials, showing substantial efficacy in removing senescent cells and decreasing inflammation levels in humans [368]. However, it has been demonstrated that the depletion of senescent β-cells results in type II diabetes in mouse models[369], indicating that high specificity is required for depletion targeting and the importance of cell type selection in treating different diseases, especially in age-related pathologies, where the senescent cell clearance may even promote the disease progression.

In addition to senolytics, which eliminate senescent cells directly, senomorphics are small molecules suppressing their SASP secretions [370]. Senomorphics mainly target pathways associated with SASP expression, including p38MAPK, PI3k/Akt, mTOR, JAK/STAT, and NF-κB [371]. The FDA-approved drugs, such as metformin inhibiting NF-κB, ruxolitinib suppressing JAK1/2, and glucocorticoids (e.g. cortisol and corticosterone) against IL-1α/ NF-κB signaling, can be used as potential senomorphics [365]. Meanwhile, antibodies blocking surface receptors for suppressing senescence or targeting SASP components, such as IL-8, IL-6, and IL-1β, for neutralizing the function and activity of SASP can also be used as senomorphics [372]. However, senomorphics require an extended timeframe to reach their optimal performance, and their interruption in gene expression also affects other related critical pathways, which possess higher risk than senolytics [365]. Although senotherapeutics present a promising approach to improving health, further studies are required to enhance their effectiveness without compromising the positive effects of cellular senescence. Additionally, due to the high heterogeneity in cellular senescence under different disease conditions [373], it is critical to design and precisely target specific senescent cells and their secretions according to the tissue region and disease status. Therefore, customized senotherapeutic interventions are also required.

## 5. Perspectives

Macrophages are integral to immune surveillance and maintaining local homeostasis, with a significant role in regulating neuroimmune responses related to brain barriers and contributing to the immunomodulation of neuropathology. Recent multi-omics technology advances have unveiled that macrophage subpopulations in CNS regions are intricately associated with pathogenesis and disease progression. These cells exhibit diverse functions in the progression of age-associated CNS conditions. Strategic approaches targeting macrophages, such as macrophage depletion, subtype reprogramming, signaling regulations, and mitochondrial transfer, offers promising avenues for suppressing disease progression in aging. While manipulating macrophage subtypes and activities holds potential for CNS disease treatments, challenges remain in enhancing recovery efficiency post CNS damage. Additional research is required to fully elucidate the functional roles of distinct macrophage subpopulations, as well as the potential adverse effects resulting from the depletion or re-polarization of macrophages. This includes investigating any abnormal cellular processes or dysregulation that may lead to cell death or tissue injury. Additionally, incorporating macrophage-targeted therapeutics with micro-based drug delivery systems enables effective and precise targeting of macrophage subtypes in treating CNS diseases. Nonetheless, micro-based drug delivery system presents significant challenges in balancing key considerations, especially delivery efficacy, binding affinity, cost, and drug loading capacity.

Moreover, limitations in current experimental techniques and the phenotypic similarities between macrophage subsets have hindered the ability to demarcate their discrete roles and contributions to disease pathogenesis. This knowledge gap regarding the heterogeneity and specialization of macrophage/ microglial populations represents a critical barrier to understanding the precise mechanisms by which these cells influence CNS-related pathologies, subsequently affecting the development of novel targeted therapeutics. The lack of selective biomarkers for distinguishing macrophages of different subtypes poses as the major hurdle. Identifying specific markers for precise and effective targeting of macrophage subtypes could enhance drug delivery efficacy to specific macrophages in regions of interest, fostering the development of more effective and selective therapies for treating different CNS conditions.
